# Health-related quality of life in COVID-19 patients: a systematic review and meta-analysis of EQ-5D studies

**DOI:** 10.1186/s12955-025-02421-8

**Published:** 2025-10-07

**Authors:** Kidu Gidey, Yirga Legesse Niriayo, Solomon Weldegebreal Asgedom, Erica Lubetkin

**Affiliations:** 1 Department of Clinical Pharmacy, School of Pharmacy, College of Health Sciences, Mekelle University, Mekelle, Ethiopia; 2 Department of Community Health and Social Medicine, CUNY School of Medicine, New York, NY USA

**Keywords:** COVID-19, HRQoL, Health-Related quality of life, EQ-5D, Systematic review

## Abstract

**Background:**

COVID-19 has affected millions globally, with a significant proportion experiencing long-COVID and impaired health-related quality of life (HRQoL). This systematic review and meta-analysis aimed to synthesize the existing literature on HRQoL in COVID-19 patients.

**Methods:**

We conducted a systematic search of PubMed, Embase, Web of Science, Scopus, and the Cochrane Library for studies published between December 2019 and March 2025. Eligible studies were peer-reviewed and assessed HRQoL in COVID-19 patients using the EQ-5D instrument. Study quality and risk of bias were evaluated using the Newcastle-Ottawa Scale. Pooled health utility values were estimated using a random-effects model, and heterogeneity was assessed via I^2^ statistics. Predictors of poor HRQoL were qualitatively narrated.

**Results:**

Out of 3539 references, 187 studies with 116,525 participants were analyzed. The majority (80.2%) used the EQ-5D-5 L version. The pooled mean EQ-5D utility score was 0.76 (95% CI 0.74–0.79, I^2^ = 99.9%) while the mean EQ-5D Visual Analogue Scale (VAS) score was 70.76 (95% CI 68.48–73.04; I^2^ = 99.7%). Pain/discomfort and anxiety/depression were the most affected domains, reported by 51% and 46% of patients, respectively. Subgroup analysis showed significant differences in HRQoL based on national income status (*p* = 0.038) and geographic region (*p* < 0.001). Common predictors of lower HRQoL included older age, female gender, disease severity, comorbidities, and post-COVID-19 symptoms.

**Conclusion:**

This systematic review demonstrates a substantial reduction in HRQoL among COVID-19 patients compared to the general population. The pooled utility values of COVID-19 contribute to understanding patients’ HRQoL and can assist in calculating Quality-Adjusted Life Years. This provides essential data for future economic evaluations and informs health policy decisions.

**Supplementary Information:**

The online version contains supplementary material available at 10.1186/s12955-025-02421-8.

## Introduction

 COVID-19 was discovered in December 2019 in Wuhan, China. Thereafter, the virus continued to spread, affecting millions of individuals across the world [1]. COVID-19 presents a wide spectrum of symptoms, ranging from mild to life-threatening, with many patients experiencing prolonged effects known as long COVID [2–4]. As a result of the persistent symptoms and complications, people with long COVID face significant reductions in health-related quality of life (HRQoL) [5, 6]. Therefore, HRQoL has become a crucial outcome measure for understanding the broader impact of COVID-19.

A variety of validated surveys exist for measuring a patient’s HRQoL, which mainly include the EQ-5D, Health Utilities Index (HUI), and Short-Form 6-Dimension (SF-6D) questionnaires [7]. The EQ-5D is the most widely used HRQoL instrument due to its robustness, reliability, and responsiveness across many health conditions and countries [8]. It is also a preferred method for evaluating health state utilities involved in health technology assessment as recommended by National Institute for Health and Care Excellence (NICE) [9]. Compared with instruments such as the SF-36 or HUI, the EQ-5D is well suited for COVID-19 research due to its extensive validation, brevity, and ease of use, making it particularly appropriate for rapid evaluation in both clinical and community settings during a pandemic when timely measurement is critical [10]. However, EQ-5D may not fully capture some aspect of quality of life such as fatigue, cognitive dysfunction, and breathlessness which are common symptoms of long-COVID not explicitly represented in its five dimensions [11].

The EQ-5D questionnaire consists of two components: the descriptive system and the visual analogue scale (EQ-VAS) [12]. The descriptive system assesses the current self-reported health status in five dimensions: mobility, self-care, usual activities, pain/discomfort, and anxiety/depression. The EQ-5D dimensions, such as mobility and self-care, are critical for assessing COVID-19’s impact. Both acute and long COVID often impair physical functioning and daily independence more abruptly than many chronic conditions, and studies report significant declines in these symptoms among affected patients [13].

The EQ-VAS evaluates the overall health status of the respondent on a visual analogue scale ranging from 0 (worst imaginable health) to 100 (best imaginable health). Two versions of the EQ-5D are available: the EQ-5D-3 L and the EQ-5D-5 L. The key difference between these versions lies in the descriptive system, with the EQ-5D-3 L having 3 levels of severity for each dimension (no problems, some problems, extreme problems) and the EQ-5D-5 L offering 5 levels (adding mild and severe problems) to provide greater sensitivity. The EQ-5D index is calculated by applying a ‘value set/tariff’ to each health profile, converting the descriptive data into a single summary score. These tariffs are derived from population-based surveys and are specific to individual countries or regions, reflecting the societal preferences for different health states [14]. The recall period for EQ-5D is today [15].

The HRQoL measured by EQ-5D plays a crucial role in economic evaluation [8] Systematic reviews and meta-analyses, considered the highest level of evidence, enhance the precision and generalizability of EQ-5D utility values by employing formal synthesis techniques that pool data from multiple studies [16]. As economic analysts increasingly rely on these comprehensive reviews to inform decision-analytic models, experts advocate for transparent and high-quality meta-analyses to strengthen the evidence base, ensuring better decision-making and outcomes in economic evaluations [17, 18].

Understanding the impact of COVID-19 on HRQoL is crucial for healthcare professionals and policymakers in developing effective interventions. Long COVID has emerged as a significant public health concern globally, defined by the World Health Organization as persistent symptoms occurring at least three months after the initial SARS-CoV-2 infection onset, lasting for at least two months, and not explained by an alternative diagnosis [19]. Millions of people have been affected with long COVID since the beginning of the pandemic with substantial implications for HRQoL [20]. While numerous studies have investigated the impact of COVID-19 on HRQoL, a comprehensive analysis particularly using the EQ-5D is still lacking. Existing systematic reviews have documented significant HRQoL reductions among COVID-19 survivors; however, some did not report utility values [21] which are essential for economic evaluation, while others relied on a variety of measurement tools and included only a small number of studies conducted during the early stages of the pandemic [22, 23]. In addition, there are several articles that have been published since the last reviews, and a comprehensive analysis would be beneficial to provide a pooled utility values and with extensive subgroup analysis. This study aimed to systematically review the available evidence on COVID-19 patients’ HRQoL using the EQ-5D, estimate pooled utility values, identify key influencing factors, explore subgroup differences, and assess the most affected EQ-5D dimensions.

## Materials and methods

### Study design

This review was developed in accordance with Preferred Reporting Items for Systematic Reviews and Meta-Analyses (PRISMA) guidelines (Appendix 1) [24]. Protocol registration was not undertaken for this systematic review, as our aim was not to estimate treatment effects.

### Search strategy

A systematic search of all studies published between December 1, 2019, and March 1, 2025, was conducted in PubMed, Embase, Web of Science, Scopus, and Cochrane Library. We developed search strings using a combination of keywords related to the EQ-5D and COVID-19. The complete search terms for each database are provided in Table S1. In addition, we systematically hand-searched the reference lists of all included studies and relevant reviews to identify any additional eligible articles not captured through electronic database searches. However, gray literature was not included, as we focused on peer-reviewed publications to ensure consistency and reliability in study quality.

### Eligibility criteria

#### Inclusion criteria

Studies were eligible for inclusion if they met the following criteria:

- Report on HRQoL in patients with COVID-19.
- Reported either or both EQ-5D index values and EQ-VAS scores using the EQ-5D-3 L or EQ-5D-5 L instruments involving adult populations.
- Were reported in English.

#### Exclusion criteria

Studies were excluded if:

- They were editorial, letter to the editor, comment, narrative case report, or opinion article.
- They were a review, intervention or a protocol.
- The EQ-5D outcomes (utility values or EQ-VAS) were not reported.
- Studies reported EQ-5D values derived from mapping other measures of health outcomes.
- No relevant data were presented (e.g. only abstract is available or full text not available).

### Study selection

The titles and abstracts of all identified studies were exported into EndNote (version 20.0.1, 2020 Clarivate), a reference management software, and duplicates were removed. The titles and abstracts of articles from the electronic database searches were independently screened by two reviewers using the pre-specified eligibility criteria (first screening). After the title/abstract review, full-text articles were reviewed by two reviewers to evaluate the eligibility of studies for inclusion (second screening). Discrepancies regarding study inclusion were resolved through consensus with a third author.

### Risk-of-bias (quality) assessment

Quality was assessed using the Newcastle-Ottawa scale (NOS) for cohort and case-control studies [25] and the adapted NOS tool for cross-sectional studies [26] (Appendix 2). Two reviewers independently assessed the risk of bias/quality appraisal. Any discrepancies were solved by a discussion or by consulting a third researcher. Judgments on the overall risk of bias were categorized as either low, moderate, or high risk based on the quality score.

### Data extraction

Data extraction was performed by two independent researchers using a data extraction form prepared on Microsoft Excel (Version 2016). For each included study, the following data were extracted:

- Publication details: authors, publication date, country/countries where study was conducted.
- Study design.
- Number of participants, mean age, percentage of males and females.
- Socio-demographic characteristics (educational status, income level, employment status, smoking status, residence (urban or rural)).
- Location of the participants (e.g. ICU, inpatient (wards), community).
- Version of EQ-5D descriptive system (e.g., EQ-5D-3 L, EQ-5D-5 L).
- Mode of administration of the EQ-5D instruments (self-complete, interviewer-administered, digital).
- Response rate in percentage.
- Mean duration of COVID-19 at the time of assessment of EQ-5D instruments.
- Comorbidities (percentage of patients with hypertension, diabetes mellitus, asthma, COPD, coronary artery disease, kidney disease, malignancy, HIV/AIDS).
- Severity of the disease (asymptomatic, mild, moderate, severe).
- Tariff used.
- EQ-5D index scores (5 L and 3 L).
- EQ-VAS scores.
- EQ-5D health profiles [Percentage reporting no problem in each dimension].

There were many rounds of scientific meetings among the three investigators for consensus on any differences in article screening, selection, and data extraction.

### Statistical analysis

Data were recorded using a Microsoft Excel sheet (version 16). All statistical analyses were performed using R version 4.3.3 (R Foundation for Statistical Computing, Vienna, Austria). The selected studies were reviewed, and the pooled estimate of health utility values (the means of EQ-5D utility scores and EQ-VAS scores) were computed by a Random effect model meta-analysis (DerSimonian–Laird method). Heterogeneity among included studies was assessed using the I^2^ statistics [27]. We conducted sensitivity analyses to assess the robustness of the results, and Egger’s regression test was used to examine publication bias.

Most studies reported the mean health utility values. For studies presenting median values with interquartile ranges or overall ranges, we estimated corresponding means and standard deviations using established techniques [28, 29]. However, these methods are primarily established under the assumption of normal data and perform poorly for skewed distributions, potentially leading to biased or less accurate estimates [29]; this limitation should be considered when interpreting the pooled estimates. In cases where EQ-5D utility scores and VAS scores were measured at multiple time points; we used the data from the final assessment. Depending on the availability of data, subgroup analyses were performed using the following covariates: age group (e.g., over 60 years old, under 60 years old), study design, timing of HRQoL measurements after COVID-19 diagnosis (HRQoL at < 12 weeks vs. HRQoL at ≥ 12 weeks), geographical regions, low-middle income vs. high-income countries, and version of the EQ-5D instruments (3 L vs. 5 L).

## Results

### Study selection

**Fig. 1:**
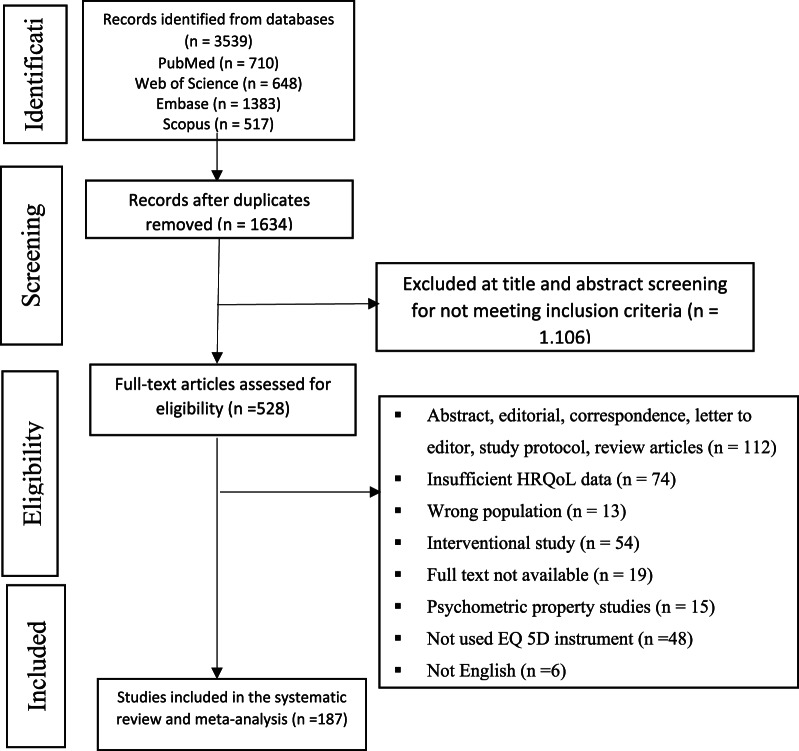
Flow diagram showing the selection process of primary studies

A total of 3539 references were initially retrieved from the databases. Following the removal of duplicates, 1905 records underwent further evaluation through title and abstract screening. A full-text examination was then performed on 528 studies, leading to the selection of 187 studies for systematic review and meta-analysis. No further relevant studies were identified through manual searching. Study selection process is illustrated in a PRISMA flow diagram shown in Fig. 1.

### Basic characteristics of the included studies

The systematic review analyzed 187 studies involving 116,525 participants ranging from 10 to 19,784. The mean age of participants was 52.6 years (SD: 9.8), and males comprised 42.3% of the population. Most studies were conducted in Europe (56.1%), followed by Asia (19.3%) and North America (11.2%), with fewer studies conducted in South America (5.9%), Africa (2.7%), and Australia (2.7%). Only four studies involved more than one continent. The study designs were mainly cohort studies (58.3%, *n* = 109), followed by cross-sectional surveys (40.1%, *n* = 75) and case-control studies (1.6%, *n* = 3) (Table S2).

### Health-related quality of life evaluation methods

The majority of the studies included (80.2%) used the EQ-5D-5 L version. The most frequent mode of administration was via face-to-face interviews (*n* = 44, 23.5%) followed by digital (*n* = 43, 23%). Approximately 68% of the studies reported EQ-5D index values, while 86.1% provided EQ-5D VAS scores. The UK (*n* = 20) and US (*n* = 12) value sets were the most applied across the studies, and 75 of the studies did not report which value set they had used. Additionally, EQ-5D profiles were presented in more than half of the studies (55.6%) (Table 1).

**Table 1:** HRQoL in patients with COVID-19 disease measured by the EQ-5D

Author, publication year	Version of EQ-5D	Utility score			VAS scores		Percent reporting “no problem” in 5 dimensions					Administration
		Mean	SD	Value set	Mean	SD	MO	SC	UA	PD	AD	
Arab-Zozani et al., 2020 [30]	EQ-5D-5 L	0.6125	0.006	Iranian	–	–	53.34	87.75	58.97	57.97	41.26	Telephone interview
Daher et al., 2020 [2]	EQ-5D-5 L	–	–	–	63*	53–80	–	–	–	–	–	Self-administered
Betschart et al., 2021 [112]	EQ-5D-5 L	–	–	–	75	19	70	92	68	45	68	Self-administered and mail
Meys et al., 2020 [113]	EQ-5D-5 L	0.62	0.19	Dutch	50.71	18.87	21	85.7	6.2	2.9	30	Digital
Fernandes et al., 2021 [114]	EQ-5D-5 L	–	–	–	75	60–90	86.7	89.6	53.1	68.7	62.5	Telephone interview
Halpin et al., 2021 [115]	EQ-5D-5 L	0.724	0.223	UK	–	–	69.1	82.4	63.2	85.3	83.8	Telephone interview
Hodgson et al., 2021 [116]	EQ-5D-5 L	0.8*	0.7–0.9	Australian	70*	60–85	57.3	84.3	55.7	50.4	60	Telephone interview
Huang et al., 2021 [53]	EQ-5D-5 L	–	–	–	80*	75–90	94	99	98	73	77	Face-to-face interview
Iqbal et al., 2021 [71]	EQ-5D-5 L	–	–	–	70.76	22.42	50	69.6	27.2	25.9	39.9	Telephone interview
Johnsen et al., 2021 [117]	EQ-5D-5 L	0.74	0.15	–	70*	55–81	–	–	–	–	–	Face-to-face interview
Kaso et al., 2021 [31]	EQ-5D-3 L	0.688	0.285	Zimbabwe	69	12.9	–	–	–	–	–	Face-to-face interview
Kohlbrenner et al., 2021 [76]	EQ-5D-5 L	0.96*	0.82, 1.00	German	80	74,94	–	–	–	–	–	Digital
Kotwani et al., 2021 [118]	EQ-5D-5 L	0.755	0.18	Thailand	75.05	12.12	43.5	57.4	52.8	41.7	17.6	Telephone interview
Lerum et al., 2021 [119]	EQ-5D-5 L	0.72	0.19	UK	–	–	60	80	40	60	60	Digital
Malinowska et al., 2021 [32]	EQ-5D-5 L	–	–	–	64.83	18.6	76.1	89.5	73.1	73.1	88.1	Telephone interview
Menges et al., 2021 [120]	EQ-5D-5 L	0.89	0.16	Dutch	85	14	88.8	99.5	89.5	64.7	69.1	Digital
Monti et al., 2021 [121]	EQ-5D-3 L	–	–	–	74	16	82	85	78	54	79	Telephone interview
Och et al., 2021 [122]	EQ-5D-5 L	–	–	–	64.4	16.2	38.4	68.5	50.7	71.2	72.6	Telephone interview
Ordinola Navarro et al., 2021 [101]	EQ-5D-5 L	–	–	–	85*	75–90	73	92	62	40	48	Self-administered
Ozkeskin et al., 2021 [123]	EQ-5D-3 L	0.81	0.19	UK	80.5	20.1	–	–	–	–	–	Digital
Rousseau et al., 2021 [124]	EQ-5D-3 L	–	–	–	71*	61–80	–	–	–	–	–	Face-to-face interview
Shah AS et al. 2021, [125]	EQ-5D-5 L	0.9*	0.81–0.95	Canadian	80*	75–90	–	–	–	–	–	Self-administered
Shah et al., 2021 [54]	EQ-5D-3 L	–	–	–	55.83	22.94	43.8	77.6	20.5	18.9	31.3	Digital
Tessitore et al., 2021 [126]	EQ-5D-5 L	–	–	–	78.4	16.1	90	98	79	58	55	Telephone interview
Todt et al., 2021 [55]	EQ-5D-3 L	0.80*	0.74-1.00	Brazilian	–	–	86	92.6	84.4	60.5	65.6	Telephone interview
Walle-Hansen et al., 2021 [33]	EQ-5D-5 L	–	–	–	65.8	19.1	90	83	89	67	74	Self-administered
Akova et al., 2022 [127]	EQ-5D-5 L	–	–	–	83.9	16.1	73.5	94.7	82.1	60.9	61.6	Self-administered
Attauabi et al., 2021 [128]	EQ-5D-5 L	0.86	0.18	Danish	77.1	18.8	75.4	89.4	64.9	54.4	51.8	Self-administered
Azizi et al., 2022 [34]	EQ-5D-5 L	–	–	–	50.89	20.46	13.8	16.4	11.3	9.3	5.4	Telephone interview
Barani et al., 2022 [67]	EQ-5D-5 L	0.925	0.15	Thailand	90.68	11.81	94.1	97.8	91.7	84.4	87.6	Telephone interview
Barreto et al., 2022 [56]	EQ-5D-5 L	–	–	–	70*	50–80	–	–	–	–	–	Face-to-face interview
Cuerda et al., 2022 [129]	EQ-5D-5 L	–	–	–	40*	25–50	15	39	10	36	66	Face-to-face or telephone interview
d’Ettorre et al., 2022 [35]	EQ-5D-5 L	0.79	0.26	Italian	72.38	15.18	66.4	86.1	62.1	45.2	48.9	Telephone interview
Oliveira et al., 2022 [130]	EQ-5D-3 L	–	–	–	80*	70–100	70.9	93.4	67.1	53.5	50.2	Telephone interview
Demoule et al., 2022 [66]	EQ-5D-3 L	0.91*	0.52-1.00	French	70*	60–85	71	79	76	66	53	Face-to-face or telephone interview
Farhanah et al., 2022 [131]	EQ-5D-5 L	0.92	0.098	Indonesian	82.98	11.56	89.4	91.3	68.3	82.7	72.1	Telephone interview
Fontes et al., 2022 [132]	EQ-5D-5 L	0.66	0.26	Portuguese	65	21	38.4	63.7	22.2	43.4	35.4	Telephone interview
Haberland et al., 2022 [133]	EQ-5D-5 L	0.92	0.12	German	83.6	15.2	–	–	–	–	–	Self-administered
Han et al., 2022 [134]	EQ-5D-5 L	0.9	0.16	US	78	18	–	–	–	–	–	Digital
Hegde et al., 2022 [36]	EQ-5D-5 L	0.95	0.2	US	–	–	95.1	98.4	96.7	95.9	99.2	Telephone interview
Heubner et al., 2022 [135]	EQ-5D-5 L	–	–	–	60*	45–75	–	–	–	–	–	Telephone interview
Huynh et al., 2022 [37]	EQ-5D-5 L	0.86	0.21	Vietnam	78.6	19.9	80	93.5	83.4	54.8	53.5	Self-administered
Kalyani et al. 2022 [136]	EQ-5D-5 L	0.88	0.12	UK	72.5	15.3	–	–	–	–	–	Self-administered
Kaso et al., 2022 [38]	EQ-5D-5 L	0.94*	0.78–0.97	Ethiopian	87*	70, 91	59.6	59.4	50.9	40.4	45.4	Telephone interview
Koullias et al., 2022 [39]	Equation 5D5L	0.788	0.244	–	73.78	17.65	–	–	–	–	–	Self-administered
Lim et al., 2022 [88]	EQ-5D-3 L	0.881	0.147	Canadian	78.8	18.1	85	96	85	64	68	Mail
Luong et al., 2022 [137]	EQ-5D-5 L	0.84	0.13	US	0.74	0.19	–	–	–	–	–	Mail
Martins et al., 2022 [138]	EQ-5D-5 L	–	–	Portuguese	75*	40–100	37	70	34	30	0	Telephone interview
Moens et al., 2022 [139]	EQ-5D-3 L	0.57	0.23	Belgian	56.6	18.2	57.04	86.84	17.55	10.42	57.4	Digital
Morrow et al., 2022 [140]	EQ-5D-5 L	0.77	0.23	UK	–	–	43.8	–	–	–	–	Digital
Nakshbandi et al., 2022 [141]	EQ-5D-5 L	0.83	0.13	Dutch	79.8	15.7	–	–	–	–	–	Digital
Ojeda et al., 2022 [98]	EQ-5D-5 L	0.8*	0.57–0.87	Spanish	70*	60–80	30.8	67.7	53.8	40	58.5	Self-administered
Pacho-Hernández et al., 2022 [99]	EQ-5D-5 L	0.8	0.2	Spanish	–	–	–	–	–	–	–	Face-to-face interview
Pan et al., 2022 [142]	EQ-5D-5 L	0.9	0.12	China	–	–	–	–	–	–	–	Face-to-face interview
Said et al., 2022 [57]	EQ-5D-5 L	0.81	0.14	US	73	21	–	–	–	–	–	Digital
Schallner et al., 2022 [143]	EQ-5D-5 L	0.32	0.4	German	30	35.3	–	–	–	–	–	Self-administered
Soh and Cho, 2022 [58]	EQ-5D-5 L	0.94	0.09	Korean	–	–	93.2	98.6	85	76.2	74.1	Digital
Tabacof et al., 2022 [144]	EQ-5D-5 L	–	–	–	64*	6–99	44	15	19	72	17	Digital
Tak, 2023 [89]	EQ-5D-5 L	0.51	0.31	US	41.6	21.1	37.4	59	3.7	12.2	28.1	Self-administered
Tarazona et al., 2022 [83]	EQ-5D-5 L	0.87	0.19	French	78	17.6	–	–	–	–	–	Telephone interview
Tsuzuki et al., 2022 [90]	EQ-5D-3 L	0.91	0.17	Japanese	80	16.8	–	–	–	–	–	Mail
Umbrello et al., 2022 [80]	EQ-5D-5 L	0.798	0.288	Italian	80*	60–89	49	77	52	48	67	Telephone interview
Vejen et al., 2022 [145]	EQ-5D-5 L	–	–	–	75*	50–90	49	77	46	39	60	–
Weihe et al., 2022 [146]	EQ-5D-5 L	–	–	–	70*	51–80	–	–	–	–	–	Telephone interview
Wimmer et al., 2022 [147]	EQ-5D-5 L	0.749	0.176	German	67.4	16.6	23.7	45.8	45.8	23.7	74.6	Face-to-face interview
Wu D, et al. 2022 [148]	EQ-5D-5 L	0.88	0.15	Chinese	80.9	14.2	70	90	70	50	50	Digital
Zhang et al., 2022 [40]	EQ-5D-5 L	0.94	0.12	Chinese	81.89	14.76	91.37	98.04	93.33	79.61	68.24	Face-to-face or telephone interview
Fietsam et al., 2023 [149]	EQ-5D-5 L	–	–	–	68.9	18.4	–	–	–	–	–	Self-administered
Hoque et al., 2023 [41]	EQ-5D-5 L	0.78	0.19	US	70.26	11.13	54.23	49.01	47.21	44.86	37.84	Face-to-face interview
Huarcaya-Victoria et al., 2022 [6]	EQ-5D-5 L	–	–	–	80*	70–90	71.4	67.2	85.7	40.3	58	Telephone interview
Iribarren-Diarasarri et al., 2022 [79]	EQ-5D-5 L	0.709	0.247	Spanish	60.53	17.6	53.9	79.7	55.3	69.3	72.7	Face-to-face interview
Román-Montes et al., 2023 [72]	EQ-5D-5 L	–	–	–	80*	70–90	69	86	66	40	47	Digital
Rosa et al., 2023 [73]	EQ-5D-3 L	0.8	0.24	Brazilian	–	–	–	–	–	–	–	Telephone interview
Rousseau et al., 2023 [150]	EQ-5D-3 L	0.7	0.2	Belgian	71.3	18.8	–	–	–	–	–	Face-to-face interview
Sánchez-García et al., 2023 [151]	EQ-5D-5 L	–	–	–	60.6	20.2	62.5	67.9	50	44.6	64.3	Self-administered
Shah et al., 2023 [42]	EQ-5D-3 L	–	–	–	91.69	12.34	17	18.6	17	20.4	17.5	Telephone interview
Wong et al.,0.2023 [94]	EQ-5D-5 L	–	–	–	60*	50–75	–	–	–	–	–	Self-administered
Taboada et al., 2020 [43]	EQ-5D-3 L	0.7054	0.25	Spanish	66.36	18.26	44	87	63	52	54	Face-to-face interview
Ferrarello et al., 2023 [91]	EQ-5D-3 L	0.87	0.16	Italian	80	19	64	86	68	59	64	Face-to-face or telephone interview
Slotegraaf et al., 2023 [59]	EQ-5D-5 L	–	–	–	67.9	19.1	–	–	–	–	–	Digital
Zupanc et al., 2023 [152]	EQ-5D-3 L	0.61	0.36	Slovenian	80	14	39	71	39	55	88	Self-administered
Giurgi-Oncu et al., 2021 [74]	EQ-5D-5 L	–	–	–	64	14.6	–	–	–	–	–	Self-administered
Cavalleri et al., 2022 [153]	EQ-5D-3 L	–	–	Belgian	72.5*	60–80	–	–	–	–	–	Telephone interview
Sandmann et al., 2021 [44]	EQ-5D-5 L	0.91	0.18	UK	80.2	18.4	85.8	94.8	83.9	61	59.9	Digital or mail
Carenzo et al., 2021 [154]	EQ-5D-5 L	–	–	–	85*	77.5–90	81	100	70	55	60	Face-to-face or telephone interview
Morelli et al., 2022 [155]	EQ-5D-5 L	–	–	–	70.4	18.3	–	–	–	–	–	Face-to-face interview
Evans et al., 2022 [45]	EQ-5D-5 L	0·75	0.22	UK	67·0	21	46	74	43	44	51	Self-administered
Brus et al. 2023 [52]	EQ-5D-5 L	0.52	0.28	Dutch	47.2	18.7	39.8	82.3	29	29.4	52.8	Digital
Sawano et al. 2025 [156]	EQ-5D-5 L	–	–	–	49*	32–61	–	–	–	–	–	Digital
Scott et al. 2023 [157]	EQ-5D-5 L	0.7	0.29	US	65.7	21	65	88	50	45	50	Digital
Khoja et al. 2024 [158]	EQ-5D-5 L	0.49	0.2	UK	44	15	40	93.3	6.7	3.3	6.7	Face-to-face interview
Cataldo et al. 2024 [159]	EQ-5D-5 L	–	–	–	65.4	17.4	66.1	89.9	51.4	24.8	12.8	Face-to-face interview
Wemhöner et al. 2025 [160]	EQ-5D-5 L	–	–	–	60.4	19.9	–	–	–	–	–	Face-to-face interview
Engel et al. 2025 [161]	EQ-5D-5 L	–	–	–	64*	50–80	–	–	–	–	–	Face-to-face interview
Malesevic et al. 2023 [162]	EQ-5D-5 L	0.74	0.21	German	59	20.3	–	–	–	–	–	Mail
Ding et al. 2024 [163]	EQ-5D-5 L	0.92	0.14	Chinese	–	–	–	–	–	–	–	Digital
Crescioli et al. 2024 [164]	EQ-5D-5 L	0.61*	0-0.91	Danish	50	0–80	55	70	50	40	55	Telephone interview
Egger et al. 2024 [85]	EQ-5D-5 L	0.63	0.33	German	59	23.9	32.3	59.7	22.6	16.1	45.2	Face-to-face or telephone interview
Neelima et al. 2023 [81]	EQ-5D-5 L	0.51	0.43	Indian	68.97	22.27	23.4	32.7	43	30.8	26.2	Face-to-face interview
Leavy et al. 2024 [165]	EQ-5D-5 L	0.72	0.25	UK	–	–	–	–	–	–	–	Face-to-face interview
Berentschot et al. 2024 [60]	EQ-5D-5 L	0.8	0.22	–	73.4	18.2	–	–	–	–	–	Mail
Zhao X, et al. 2024 [166]	EQ-5D-5 L	–	–	–	78.76	9.18	86.1	93.6	90.2	95.4	90.2	Face-to-face interview
Colleran R et al. 2023 [167]	EQ-5D-5 L	–	–	–	80	14.8	85	98	80	70	75	Face-to-face or telephone interview
Visser et al. 2024 [77]	EQ-5D-5 L	0.74	0.19	Dutch	65.8	18.6	–	–	–	–	–	–
Neumann et al. 2025 [46]	EQ-5D-5 L	0.91*	0.89-1.00	–	–	–	–	–	–	–	–	–
Appel et al. 2024 [168]	EQ-5D-5 L	0.8	0.2	–	73.9	20.3	–	–	–	–	–	Face-to-face or telephone interview
Deesomchok et al. 2023 [169]	EQ-5D-5 L	0.78	0.18	Thailand	81.8	11.6	–	–	–	–	–	–
Kato et al. 2025 [170]	EQ-5D-5 L	–	–	Japanese	80*	65, 90	–	–	–	–	–	Mail
Rego de Figueiredo et al. 2023 [47]	EQ-5D-5 L	0.73	0.26	Portuguese	64	22	–	–	–	–	–	Mail
Gorsler et al. 2024 [171]	EQ-5D-5 L	0.8	0.2	–	67.4	17.1	–	–	–	–	–	Face-to-face interview
Naik et al. 2025 [87]	EQ-5D-5 L	0.81	0.15	Canadian	68.8	17.2	85.3	93.7	75.5	63.5	50.9	Digital
Sun X et al. 2024 [100]	EQ-5D-5 L	0.86	0.17	US	–	–	–	–	–	–	–	Digital
Hatakeyama et al. 2025 [48]	EQ-5D-5 L	0.804	0.336	–	–	–	–	–	–	–	–	Mail
Soare et al. 2024 [75]	EQ-5D-5 L	0.84	0.22	UK	80	18.7	–	–	–	–	–	Digital
Rover et al. 2024 [172]	EQ-5D-3 L	0.84	0.15	Brazilian	–	–	–	–	–	–	–	Telephone interview
Janols et al. 2024 [173]	EQ-5D-5 L	0.77	0.22	Swedish	50	40	–	–	–	–	–	Mail
Guaraldi et al. 2023 [174]	EQ-5D-5 L	0.8	0.2	Spanish	67	16	–	–	–	–	–	Digital
Kwon et al. 2024 [175]	EQ-5D-5 L	0.501	0.287	UK	51.9	21.2	–	–	–	–	–	Digital
Smith et al. 2023 [176]	EQ-5D-5 L	0.91	0.13	Belgian	–	–	–	–	–	–	–	Digital
Tak C 2023 [177]	EQ-5D-5 L	0.51*	0.59, 039	US	41.6*	55,31	37.4	59	3.7	12.2	28.1	Digital
Qorolli et al. 2023 [178]	EQ-5D-5 L	0.7	0.2	–	66.8	16.3	35.9	79.5	25.7	30.8	76.9	Face-to-face interview
Tiels et al. 2025 [102]	EQ-5D-5 L	0.67	0.25	Dutch	60.9	16.8	–	–	–	–	–	Face-to-face interview
Hansen KS et al. 2023 [179]	EQ-5D-5 L	0.69*	0.69–0.70	–	–	–	–	–	–	–	–	Face-to-face interview
Mastrorosa et al. 2023 [61]	EQ-5D-3 L	–	–	Italian	70.1	18.8	–	–	–	–	–	Face-to-face interview
Elneima O, et al. 2024 [180]	EQ-5D-5 L	0.64	0.27	–	67.3	21.2	–	–	–	–	–	Face-to-face interview
Sun et al. 2023 [181]	EQ-5D-5 L	0.808	0.204	US	73.3	16.9	17.2	11.4	16.6	27.6	41	Digital
Fernández-de-las-Peñas et al. 2023 [182]	EQ-5D-5 L	0.75	0.25	Spain	–	–	–	–	–	–	–	Face-to-face interview
Mercier et al. 2023 [183]	EQ-5D-5 L	0.863	0.116	Canada	–	–	–	–	–	–	–	Face-to-face interview
Tsuruoka et al. 2025 [184]	EQ-5D-5 L	–	–	Vietnam	90*	80–90	94.1	95.7	94.1	82.3	77.4	–
van Tol et al. 2024 [185]	EQ-5D-5 L	0.52	0.32	Various	–	–	–	–	–	–	–	Telephone interview
Carlile et al. 2024 [68]	EQ-5D-5 L	0.49	0.31	UK	–	–	–	–	–	–	–	Digital
Carrera et al. 2025 [186]	EQ-5D-3 L	0.73*	0.59-1	–	80*	70–90	–	–	–	–	–	Face-to-face interview
Elumalai et al. 2023 [82]	EQ-5D-5 L	0.98	0.05	Indian	92.14	8.39	88.8	98.7	89.8	85	89.7	Telephone interview
Di Fusco et al. 2023 [187]	EQ-5D-5 L	0.727	0.242	US	74.5	17.2	–	–	–	–	–	Digital
Kuodi et al. 2023 [84]	EQ-5D-5 L	0.8	0.29	US	–	–	72	88	68	40	45	Digital
Godfrey et al. 2025 [188]	EQ-5D-5 L	0.53	0.19	UK	47	15	–	–	–	–	–	Telephone interview
Lomholt-Welch et al. 2023 [189]	EQ-5D-5 L	0.74	0.22	–	61.54	21.95	–	–	–	–	–	Face-to-face interview
Atchison et al. 2023 [190]	EQ-5D-5 L	0.75	0.22	UK	64.7	21.1	53.3	79.4	36.6	24.3	34.6	Telephone interview
Walker et al. 2023 [95]	EQ-5D-5 L	0.54	0.27	UK	–	–	26.9	12	36.3	3.8	5.1	Digital
Schröder et al. 2024 [62]	EQ-5D-3 L	0.66	0.23	Germany	57.6	22.2	66.2	92.6	30.5	19.1	39.8	Digital
Duwel et al. 2023 [191]	EQ-5D-5 L	–	–	–	79.6	14.5	–	–	–	–	–	Telephone interview
Pietruszka-Wałęka et al. 2024 [192]	EQ-5D-5 L	–	–	Polish	76*	69–78	41.18	94.12	70.59	35.29	41.18	Face-to-face interview
Samuelsson et al. 2025 [193]	EQ-5D-3 L	0.83	0.13	Swedish	67.6	19.2	60	90	65	31	49	Mail
Seeley et al. 2025 [194]	EQ-5D-5 L	0.67*	0.49–0.80	UK	–	–	36.4	63.6	0	12.1	24.2	Face-to-face interview and Digital
Agergaard et al. 2023 [195]	EQ-5D-5 L	0.76*	0.62–0.85	–	–	–	–	–	–	–	–	Face-to-face interview and Digital
Amedewonu et al. 2024 [92]	EQ-5D-5 L	0.815	0.12	Zimbabwe	75.6	22	74	84	73.3	67.3	66.7	Face-to-face interview
D’Souza et al. 2024 [196]	EQ-5D-5 L	0.8*	0.71, 0.86	Australian	66*	45–80	51	82	23	14	12	Digital
Carenzo et al. 2024 [197]	EQ-5D-5 L	–	–	Italian	80*	70–90	69.2	88.5	69.2	41.7	56.3	Face-to-face interview
McCarthy et al. 2024 [198]	EQ-5D-5 L	0.653	0.3	–	–	–	–	–	–	–	–	Telephone interview
Malesevic et al. 2023 [199]	EQ-5D-5 L	0.818	0.168	German	66	20.3	50	87.9	69.7	46.9	65.2	Mail
Dennis et al. 2023 [200]	EQ-5D-5 L	0.71*	0.56–0.81	–	70*	60–80	–	–	–	–	–	Digital
Cannata et al. 2023 [201]	EQ-5D-5 L	–	–	–	80*	70–90	90.9	97.3	86.4	90	69.1	Face-to-face interview
Wang J et al. 2024 [202]	EQ-5D-5 L	0.64*	0.59, 0.69	UK	–	–	–	–	–	–	–	Digital
Wangnamthip S et al. 2024 [203]	EQ-5D-5 L	0.9	0.1	Thailand	87.6	12.8	–	–	–	–	–	Telephone interview
Caamano et al. 2024 [63]	EQ-5D-5 L	0.91*	0.76-1	Spanish	70*	60–90	73.3	84.2	91.1	58.4	53.5	Telephone interview
Sinaga et al. 2023 [204]	EQ-5D-5 L	–	–	Indonesian	87.6	8.1	100	100	100	96	96	Face-to-face interview
Tabacof et al. 2023 [205]	EQ-5D-5 L	–	–	–	59*	54.9–61.4	–	–	–	–	–	Digital
Thanh HN et al. 2024 [206]	EQ-5D-5 L	0.94	0.11	Vietnamese	84.2	13.11	94.4	98.7	94.7	74.6	67.3	Digital or Telephone interview
Cijs et al. 2023 [207]	EQ-5D-5 L	0.7	0.03	German	–	–	–	–	–	–	–	Face-to-face or Telephone interview
Domazet Bugarin et al. 2023 [208]	EQ-5D-5 L	–	–	–	70*	40–80	–	–	–	–	–	Telephone interview
Cázares-Lara et al. 2024 [209]	EQ-5D-5 L	0.87*	0.80–0.94	Mexican	80*	70–85	81	94.6	87.8	68.2	55.4	Face-to-face interview
Pavithra et al. 2023 [210]	EQ-5D-5 L	–	–	–	70.7	17.2	57	70	58	35	58	Face-to-face interview
Bolgeo et al. 2024 [49]	EQ-5D-3 L	0.79	0.15	UK	66.99	21.59	55.8	59.8	50.3	49.2	57.3	Digital or Telephone interview
Salem et al. 2023 [50]	EQ-5D-3 L	0.955	0.105	Malaysia	93	11	–	–	–	–	–	Telephone interview
Honda et al. 2025 [211]	EQ-5D-5 L	0.9*	0.8-1.0	–	–	–	87.7	93.3	89.7	63.2	72.7	Digital
Gharibzadeh et al. 2024 [212]	EQ-5D-5 L	–	–	–	70*	50–80	–	–	–	–	–	Face-to-face interview
Berentschot et al. 2024 [213]	EQ-5D-5 L	0.8	0.22	–	–	–	–	–	–	–	–	Mail
Macedo Junior et al. 2024 [96]	EQ-5D-3 L	0.79*	0.74–0.85	Brazilian	80*	70–90	99.1	100	89.9	55	47.7	Face-to-face interview
Álvarez-Hernández et al. 2023 [214]	EQ-5D-5 L	0.8	0.25	Spanish	72.7	19	71.3	87.8	65.4	47.3	57.4	–
Prata TA, et al. 2024 [97]	EQ-5D-3 L	–	–	–	80*	70–90	60.2	83.3	71	41.9	50.5	Face-to-face interview
Alanazi MQ et al. 2023 [215]	EQ-5D-5 L	0.92	0.13	–	86.96	15.31	–	–	–	–	–	Face-to-face interview and Digital
Moisoglou et al. 2024 [70]	EQ-5D-3 L	0.36	0.36	Greek	54.1	21.71	–	–	–	–	–	Digital
Ramos et al. 2024 [216]	EQ-5D-3 L	0.8*	0.69-1	–	80*	70–90	–	–	–	–	–	Telephone interview
Gursoy et al. 2023 [217]	EQ-5D-3 L	–	–	–	75.42	17.39	43.4	46.9	45.5	48.3	49	Face-to-face interview
Wang R et al. 2023 [218]	EQ-5D-5 L	0.52	0.35	China	48.52	24.29	31.2	50.4	22.4	9.3	35.3	Digital
Demirhan et al. 2023 [219]	EQ-5D-3 L	0.86	0.193	–	78.84	16.15	79.3	89.8	87.2	74.5	84.2	Telephone interview
Firouzabadi et al. 2024 [51]	EQ-5D-3 L	–	–	–	76.5	26.6	61.8	95.4	86.1	86.1	55.7	–
Sun C et al. 2024 [69]	EQ-5D-5 L	0.94	0.07	Chinese	–	–	94.07	97.78	94.81	81.48	70.37	Face-to-face or telephone interview
Galanis et al. 2023 [64]	EQ-5D-3 L	0.36	0.37	Greek	54.1	21.71	20.5	62.3	19.7	20.5	13.9	Digital
Dodd et al. 2024 [78]	EQ-5D-5 L	–	–	–	82.87	12.09	–	–	–	–	–	–
Rahimi et al. 2024 [220]	EQ-5D-3 L	–	–	–	75	60–85	–	–	–	–	–	Face-to-face interview
Holland et al. 2024 [221]	EQ-5D-5 L	–	–	–	62*	3–100	76	94	63	63	29	Digital or Telephone interview
Zalaquett et al. 2024 [93]	EQ-5D-5 L	–	–	–	85*	75–90	85	96	79	38	40	Digital
Bodey et al. 2024 [222]	EQ-5D-5 L	0.53	0.29	UK	57.67	20.19	–	–	–	–	–	Mail or telephone interview
Kho et al. 2023 [223]	EQ-5D-3 L	–	–	–	80*	75–90	–	–	–	–	–	Face-to-face interview
Yalçın-Çolak et al. 2023 [224]	EQ-5D-3 L	0.76	0.19	–	69.56	19.04	–	–	–	–	–	Face-to-face or telephone interview
Kılınçarslan et al. 2023 [65]	EQ-5D-3 L	1*	0.79-1.00	UK	100*	75–100	–	–	–	–	–	Digital

“–” represents the value is not reported by the article. *Represents the utility or VAS score was reported in the median (IQR or range)

*AD* Anxiety/Depression, *EQ-5D* EuroQol 5 Dimensions, *F* Face-to-face interview, *HRQoL* Health-Related Quality of Life, *IQR* interquartile range, *MO* mobility, *PD* Pain/Discomfort, *SC* self-care, *SD* standard deviation, *UA* usual activities, *VAS* Visual Analog Scale

### EQ-5D index scores, EQ-VAS scores, and EQ-5D profiles

**Fig. 2:**
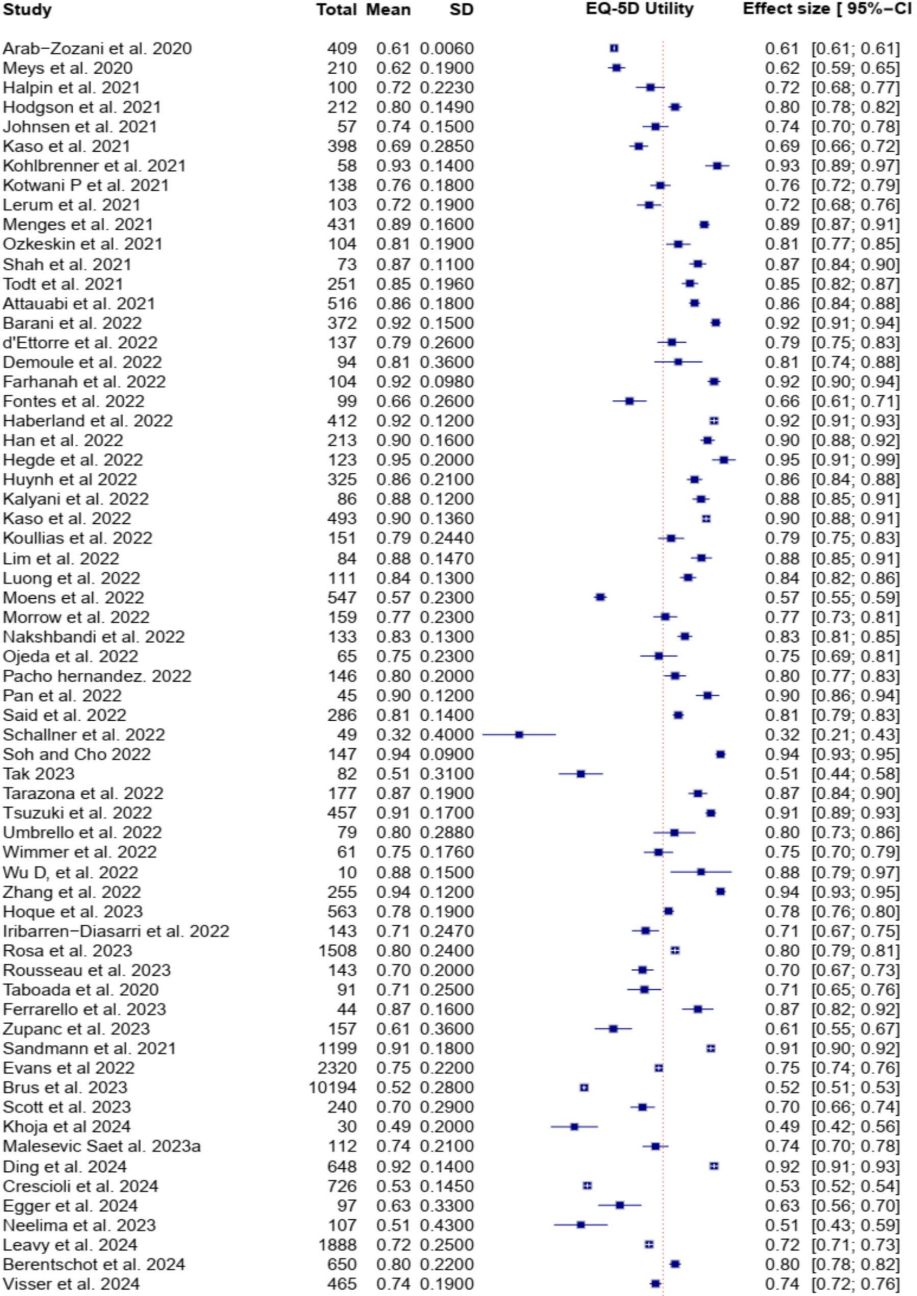
Random effect meta-analysis of EQ-5D utility in patients with COVID-19

Of the 187 studies included, 127 (68%) reported EQ-5D utility scores for COVID-19 patients, with a pooled mean EQ-5D utility score of 0.76 (95% CI 0.74–0.79) (Fig. 2). Additionally, 161 studies reported EQ-VAS scores, with a pooled mean of 70.76 (95% CI 68.48–73.04) (Table 2). Despite these pooled estimates, considerable heterogeneity was observed across studies, with an I^2^ value of 99.9% for EQ-5D index scores and 99.7% for EQ-5D VAS scores (Figure S1).

**Table 2:** Pooled estimates of EQ-5D utility scores, EQ-5D VAS scores, and EQ-5D profiles among COVID-19 patients

Variable	No. of articles	Estimate	Lower bound	Upper bound	Std. error
Pooled estimate of EQ-5D index	127	0.76	0.74	0.79	0.01
Pooled estimate of EQ-VAS Score	161	70.76	68.48	73.04	1.16
Pooled estimate of any problems in mobility (%)	104	37	32	42	2.5
Pooled estimate of any problems in self-care (%)	104	21	17	24	1.9
Pooled estimate of any problems in usual activity (%)	104	42	35	48	3.3
Pooled estimate of any problems in pain/discomfort (%)	104	51	45	56	2.8
Pooled estimate of any problems in anxiety/depression (%)	104	46	40	52	3.0

*EQ−5D* EuroQol 5 Dimensions, *VAS* Visual Analog Scale

Pooled estimates were also calculated for the different dimensions of the EQ-5D instrument, including mobility (Figure S2), self-care (Figure S3), usual activities (Figure S4), pain/discomfort (Figure S5), and anxiety/depression (Figure S6). Among these, pain/discomfort and anxiety/depression were the most affected dimensions, affecting 51% (95% CI 45–56) and 46% (95% CI 40–52) of patients, respectively, as summarized in Table 2.

### Subgroup analysis of EQ-5D utility scores

Our subgroup meta-analysis aimed to identify factors contributing to the wide variation in HRQoL among COVID-19 patients. Of the six variables examined, only geographic region and national income status showed a statistically significant association with HRQoL.

***EQ-5D instrument versions***: When comparing utility scores across the two versions of the EQ-5D instrument, the results showed that a similar mean utility score of the EQ-5D-5 L score of 0.76 (95% CI 0.74–0.79), compared to a mean score of 0.76 (95% CI 0.71–0.81) for studies using the EQ-5D-3 L version. Statistically, this difference was not significant (*p* = 0.88), as illustrated in Figure S7.

***Geographic regions***: Patients in Asia had the highest mean utility score of 0.85 (95% CI 0.76–0.93), followed by North America at 0.84 (95% CI 0.82–0.86), while Europe had the lowest at 0.72 (95% CI 0.70–0.74). The differences in mean utility scores among the different continents were statistically significant (*p* < 0.001) (Figure S8).

***Study design***: Utility scores were slightly higher in cohort studies (0.75, 95% CI 0.70–0.80) compared to cross-sectional studies (0.77, 95% CI 0.74–0.80), though the difference was not statistically significant (*p* = 0.45) (Figure S9).

***Time of HRQoL measurements after the COVID-19 diagnosis***: Patients evaluated three months or more after their diagnosis tended to report slightly higher utility scores (0.80, 95% CI 0.77–0.82) compared to those assessed within the first three months (0.78, 95% CI 0.70–0.86). However, this difference was not statistically significant (*p* = 0.72) (Figure S10).

***Income category***: When stratifying by income, patients in the low to middle-income subgroup had higher utility scores of 0.83 (95% CI 0.76–0.90) compared to the high-income subgroup at 0.75 (95% CI 0.72–0.77). This difference was statistically significant (*p* = 0.038) (Figure S11).

***Age classification***: Patients aged 60 and over tended to report slightly lower utility scores (0.74, 95% CI 0.69–0.80), than those under 60 (0.76, 95% CI 0.73–0.78), but this small difference wasn’t statistically meaningful (*p* = 0.67) (Figure S12).

### Sensitivity analysis and publication bias

We conducted a sensitivity analysis to verify the robustness of the results. The leave-one-out method showed that removing any single study did not affect the overall result, confirming that the findings are stable and not driven by any one study (Figure S13). Excluding studies with fewer than 100 participants yielded a utility score of 0.77 (95% CI 0.75–0.79). Similarly, omitting studies with a NOS score below 6 resulted in a mean utility score of 0.77 (95% CI 0.74–0.81), confirming the stability of our results (Table S3). The risk of publication bias was assessed using Egger’s regression test, and the results showed no significant evidence of bias (*p* = 0.27), indicating that the findings are likely to be reliable and not influenced by unpublished studies.

### Predictors of poor HRQoL in patients with COVID-19: qualitative synthesis of literature

 We identified 42 studies that reported on the predictors of poor HRQoL among COVID-19 survivors. We found that over 50 different predictor variables were documented (Table S4). We have grouped these predictors into three main categories: demographic, socioeconomic, and clinical factors.

### Demographic factors

Age was consistently identified as a predictor, with most studies reporting that older individuals generally exhibited lower HRQoL scores [6, 30–51]. However, one study found that younger age was linked to worse HRQoL [52]. Sex was also a significant factor; several studies consistently associated female sex with poorer HRQoL [30, 35, 37, 39, 40, 51, 53–65], whereas three studies identified male sex as a predictor of poorer HRQoL [42, 43, 66].

### Socioeconomic factors

Being unemployed was associated with poorer HRQoL [30, 35, 52, 60, 62, 67] and being a housewife or retired was also linked to lower HRQoL [41]. Higher education, compared with lower or middle education levels, was reported in some studies as a predictor of poor HRQoL [30], whereas other studies found that lower education levels were associated with worse outcomes [52, 65, 68]. Lower household income [68, 69], and living in rural areas, compared with urban settings, were also associated with poorer HRQoL [67]. Additional factors linked to reduced HRQoL included higher social impairment [62], lower resilience [70], and less support from significant others [70].

### Clinical factors

***Disease severity***: Greater disease severity during acute COVID-19 was consistently associated with poorer HRQoL [31, 33, 38, 42, 50, 58, 71–75]. Specific indicators of severe disease such as ICU admission or requirement [30, 55, 76], oxygen requirements during COVID-19 [42], lung diffusion impairment [53], pulmonary injury [74], pulmonary embolism [77], higher level of CRP levels [74, 78], and experiencing delirium [79] during the acute phase were reported to have worse HRQoL.

***Comorbidities***: Presence of comorbidity [33, 35, 37, 41, 42, 49, 52, 61, 71, 80–82], greater number of comorbidities [32, 68], pre-existing specific conditions like diabetes [30, 34, 75], cardiovascular disease [30, 34, 47, 60, 62, 74, 83], hypertension [75, 84], kidney disease [34], asthma [31, 38, 47], COPD [38, 47], pulmonary disease [60], malignancy [38], presence of metabolic diseases [58], higher preclinical frailty [85, 86], history of depression [62, 87], and autoimmune disease [62] were associated with poorer HRQoL.

***Post-COVID symptoms or syndromes***: Persistent COVID-19 symptom/long covid status [6, 37, 40, 41, 44, 58, 62, 64, 69–71, 78, 82, 83, 88–93] including specifically reported symptoms such as fatigue [46, 56, 91, 94, 95], dyspnea [56, 66, 91, 94, 96, 97], anxiety and depression [57, 58, 94, 95, 98, 99], sleep quality [99], headache [91], pain [46, 56, 57, 91, 98], memory problems [91], delirium [48], number of persistent symptoms [46, 49, 74], visual problems [65], myalgia [65], seeing a physician for olfactory dysfunction [57], disability [68], being in a higher symptom burden phenotype [100], seeking care in long COVID clinic [89], altered physical activities [101], and higher functional impairment [49] were significantly associated with poorer HRQoL.

***Hospitalization and treatment***: Duration of illness at admission [42, 54], being hospitalized [39, 84], length of hospital stay [38, 42, 43, 54, 60, 74, 80], ICU admission [51], length of ICU stay [43, 81], not being vaccinated [52, 84], hospitalization during the acute phase [75], mechanical ventilation use [43, 45, 48, 85], and duration of mechanical ventilation [63] were linked to poorer HRQoL in some studies. Other factors associated with poorer HRQoL were tracheostomy [66, 79], presence of fibrous stripe on chest CT [40], and longer duration of steroid use [42]. However, steroid use was reported in some studies to improve HRQoL compared with no steroid use [31, 61].

***Other factors*** such as obesity and/or BMI > 35 [35, 41, 45, 52, 66, 83],, history of smoking [34, 36, 53, 66, 72, 75], death of a family member from COVID-19 [6], pre-existing psychological condition [78], living alone [6, 37], stress [37], lower self-efficacy [102], and poorer HRQoL in people exercising more than 5 h per week pre-COVID compared to 2 to 5 h and 0 to 2 h [89] were also identified as predictors.

### Risk-of-bias (quality) assessment results

In our study, we utilized the NOS to evaluate the quality of included studies, focusing on three key criteria: selection, comparability, and outcome assessment. Each study was assigned a quality score out of a possible 9 points. Specifically, studies could earn up to 4 stars for selection, 2 stars for comparability, and 3 stars for outcome assessment.

We classified the quality of the articles as follows: high quality for scores between 7 and 9 stars, moderate quality for scores between 4 and 6 stars, and low quality for scores between 0 and 3 stars. The median quality score across all articles was 6, with scores ranging from 3 to 9. Among the evaluated studies, 2.7% (*n* = 5) were deemed to be of low quality, 58.5% (*n* = 110) were of moderate quality, and 38.8% (*n* = 73) were considered high quality. Detailed quality scores for each article can be found in Supplementary Table S1.

## Discussion

We conducted a comprehensive systematic review and meta-analysis of HRQoL following COVID-19, using the EQ-5D instrument for assessment. The analysis found that the pooled mean EQ-5D index score and EQ-VAS score were 0.76 and 70.76, respectively. These values indicate a reduction in HRQoL among COVID-19 patients compared to population norms. For example, reported EQ-5D utility scores from population norms in various countries, such as the US (0.87), France (0.89), and Korea (0.95), UK (0.86), China (0.95), Zimbabwe (0.83) are higher than those observed in our study [103]. A recent meta-analysis also reported a higher pooled mean EQ-5D utility score of 0.89 in the general population, further underscoring the impact of COVID-19 on patient HRQoL [104]. Lower EQ-5D utility scores in COVID-19 patients compared to population norms show the lasting impact of long COVID and the need for a holistic recovery approach. Healthcare systems should use these findings to guide comprehensive rehabilitation and mental health services aimed at restoring quality of life. Policymakers should also adopt targeted public health and socioeconomic measures to reduce disparities among vulnerable groups.

Our findings show that pain/discomfort and anxiety/depression are the most affected HRQoL dimensions, with impairments reported by 51% and 46% of participants, respectively. These results are consistent with previous research, which has also identified these dimensions as commonly affected [23]. The high prevalence of pain/discomfort may reflect persistent physical sequelae, such as musculoskeletal pain, neuropathic symptoms, and chronic fatigue, whereas anxiety/depression likely results from a combination of biological mechanisms (e.g., neuroinflammation, hypothalamic–pituitary–adrenal axis dysregulation) and psychosocial stressors (e.g., social isolation, employment loss, uncertainty about recovery) [105, 106].

We found higher levels of impairment in pain/discomfort and anxiety/depression than those reported in population norms from a multicountry European study (28.5% for pain/discomfort and 8% for anxiety/depression) [107], as well as in general population samples from China (10.7% and 8.7%) and the UK (33% and 21%) [103]. The substantial impairments in our study underscore the need for tailored clinical interventions. Implementing comprehensive pain management strategies, which include pharmacological interventions, physical therapy, and mental health support, is essential [108, 109]. Additionally, cognitive behavioral therapy and physical exercise programs have shown significant effectiveness in mitigating depression and anxiety linked to the COVID-19 pandemic [110]. Adopting these evidence-based approaches is vital for improving the overall quality of life for those affected. However, the results of our meta-analysis should be interpreted cautiously due to high study heterogeneity.

The heterogeneity in EQ-5D index scores (I^2^ = 99.9%) and VAS scores (I^2^ = 99.7%) indicates substantial variability in reported outcomes across studies. This heterogeneity may arise from various factors, including differences in study populations, geographical locations, access to rehabilitation services, and healthcare systems. Factors such as disease severity, comorbidities, socioeconomic status, and access to healthcare services can influence the variability. Moreover, variations in methodological sources including variations in EQ-5D administration (telephone, online, face-to-face), choice of value sets for index calculation, differences in time since infection when HRQoL was measured, and survey design (self-reported vs. interviewer-administered) may also contribute to the observed heterogeneity. HRQoL in COVID-19 patients may have also changed over time due to improvements in treatment, vaccination, and viral variants. These temporal factors may contribute to differences across studies and should be considered when interpreting results.

Our subgroup analysis revealed significant variations in HRQoL across geographic regions, with patients in Asia generally reporting higher utility scores (0.85) compared to those in Europe (0.72). However, direct comparisons of HRQoL across continents are inherently complex and demand cautious interpretation due to different influencing factors. These include methodological differences across studies, the specific populations surveyed (e.g., general population vs. hospitalized patients), differences in quarantine and isolation measures, diverse socioeconomic, cultural, and social conditions that impact health states. The inconsistencies warrant cautious interpretation and highlight the need for standardized EQ-5D reporting in future studies to facilitate cross-comparisons.

When stratified by income, patients in the low-middle-income subgroup reported higher utility scores (0.83; 95% CI 0.76–0.90) compared to the high-income subgroup (0.75; 95% CI 0.72–0.77). This disparity could result from the large number of articles from European countries (56%) included in the study, given that Europe generally experienced a relative decline in HRQoL, and most European countries are high-income, which could have potentially lowered the overall high-income average. The finding may also reflect differences in baseline health expectations, variations in life expectancy, differences in sample composition, or selection bias toward less severe cases in LMIC studies.

Various studies have identified predictors of poor HRQoL in COVID-19 patients, including, female gender, severe disease, comorbidities, and post-COVID symptoms, highlighting the vulnerability of certain groups. This observation is consistent with findings from a previous systematic review that employed a different HRQoL measure [111].

In our literature review, we observed that EQ-5D is widely used, but variability in the reporting of different components of the EQ-5D across various studies exists. Some studies solely reported the EQ-5D profile, while others reported either the EQ VAS or EQ-5D index, or both. For consistency and to facilitate synthesis of information, it is recommended that studies report all index scores, VAS scores, and health profiles where possible [14].

One of the strengths of our systematic review and meta-analysis is that it includes a large number of studies (187 in total) conducted over a long period. By using a single, widely accepted tool, the EQ-5D, we ensured consistent measurement of HRQoL across all studies, making it easier to combine the results. While other reviews, like the one by Nandasena et al., have also looked at HRQoL in COVID-19 patients, they only included 21 studies and did not provide a pooled estimate of utility values [111]. Malik et al. conducted a meta-analysis on post-acute COVID-19 syndrome and HRQoL, but this was done early in the pandemic and only included 12 studies [23].

Our findings have important implications for clinical practice and policy-making. They can help healthcare professionals and policymakers better understand which groups might experience a greater burden due to COVID-19. Researchers and decision-makers can also use the mean utility value from our study for cost-effectiveness analyses related to COVID-19 interventions, aiding in the calculation of QALYs. Our pooled EQ-5D utility values can inform cost–utility analyses for long-COVID programs, such as in assessing the cost-effectiveness of rehabilitation programs aimed at restoring mobility and usual activities, in prioritizing mental health interventions like cognitive behavioral therapy for anxiety/depression, and remote care models.

However, our study is not without limitations. The quality of the studies included varied, with some having small sample sizes, low response rates, or incomplete data. This variability may have affected the pooled estimates and generalizability of our findings. Additionally, we had initially planned subgroup analyses based on factors such as disease severity, complications, vaccination status, and access to COVID testing. However, due to inadequate or unclear information about these factors, we were unable to conduct these analyses. Some studies reported medians and ranges rather than means and standard deviations. To maintain consistency across studies, we used only the final HRQoL assessment when multiple time points were reported. While this approach helps standardize comparisons, it may bias results toward recovery, as HRQoL tends to improve over time. We estimated means and SDs using established methods and these estimations may be less accurate for skewed data, potentially affecting the precision of our pooled results. We also limited our review to English-language publications due to resource constraints, which may have excluded relevant studies from non-English-speaking regions.

Despite the large number of studies included, several important gaps remain that warrant attention in future research. Data on key factors such as vaccination status, which could modulate HRQoL through reduced symptom severity; access to COVID-19 testing and early diagnosis; adherence to treatment protocols like oxygen therapy or antivirals, hospitalization status or ICU admissions, limiting our ability to assess their impact on HRQoL outcomes. Future research should prioritize longitudinal studies incorporating these variables to identify modifiable risk factors and evaluate intervention efficacy over time.

## Conclusion

This systematic review and meta-analysis contribute to the growing body of literature on HRQoL in patients with COVID-19. Our findings show that individuals with COVID-19 had lower EQ-5D index scores, EQ-VAS scores, and EQ-5D profiles, indicating a reduced HRQoL compared to the general population. In our subgroup analysis, we identified geographic locations and incomes status of the countries as significant factors associated with HRQoL. The mean utility value derived from this study aids in understanding patients’ HRQoL and could assist future economic studies and policy decisions.

## Supplementary Information

Supplementary Material 1

## Data Availability

The datasets used and/or analyzed during the current study are available from the corresponding author on reasonable request.

## Abbreviations

COPD: Chronic Obstructive Pulmonary Disease
COVID-19: Coronavirus Disease 2019
EQ-5D: EuroQol-5 Dimension
EQ-VAS: EuroQol Visual Analogue Scale
HRQoL: Health-Related Quality of Life
HUI: Health Utilities Index
I^2^: I-squared (statistic for heterogeneity in meta-analysis)
ICU: Intensive Care Unit
NICE: National Institute for Health and Care Excellence
NOS: Newcastle-Ottawa Scale
PRISMA: Preferred Reporting Items for Systematic Reviews and Meta-Analyses
QALYs: Quality-Adjusted Life Years
SD: Standard Deviation
SF-6D: Short-Form 6-Dimension
