# Supplementary material for: Health-related quality of life in COVID-19 patients: a systematic review and meta-analysis of EQ-5D studies
Source: Health Qual Life Outcomes. 2025 Oct 7;23:97. doi: 10.1186/s12955-025-02421-8 (PMC12506383; doi:10.1186/s12955-025-02421-8)
Supplement: Supplementary file 1 — Supplementary Material 1 [file 12955_2025_2421_MOESM1_ESM.docx]

**Supplemental Material**

**Health-related quality of life in patients with COVID-19: a systematic review and meta-analysis of EQ-5D studies**

**Contents**

**Tables**

[Table S 1: Search strategy of databases 1](#_Toc177901854)

[Table S 2: Basic characteristics of the included studies. 4](#_Toc177901855)

[Table S 3: Sensitivity analysis 16](#_Toc177901856)

[Table S 4: Predictors of poor health related quality of life in patients with COVID-19 16](#_Toc177901857)

**Figures**

[Figure S 1: Random effect meta-analysis of EQ-VAS score in patients with COVID-19 22](#_Toc177901142)

[Figure S 2: Forest plot of pooled proportion of mobility using EQ‐5D in COVID‐19 patients. 24](#_Toc177901143)

[Figure S 3: Forest plot of pooled proportion of self-care using EQ‐5D in COVID‐19 patients. 26](#_Toc177901144)

[Figure S 4: Forest plot of pooled proportion of usual activity using EQ‐5D in COVID‐19 patients. 27](#_Toc177901145)

[Figure S 5: Forest plot of pooled proportion of pain/discomfort using EQ‐5D in COVID‐19 patients. 30](#_Toc177901146)

[Figure S 6: Forest plot of pooled proportion of anxiety/depression using EQ‐5D in COVID‐19 patients. 32](#_Toc177901147)

[Figure S 7: EQ-5D instrument versions stratified random effect meta-analysis 35](#_Toc177901148)

[Figure S 8: Geographic locations stratified random effect meta-analysis 37](#_Toc177901149)

[Figure S 9: Study design stratified random effect meta-analysis 39](#_Toc177901150)

[Figure S 10: Time of HRQoL measurements after the COVID-19 diagnosis stratified random effect meta-analysis 41](#_Toc177901151)

[Figure S 11: Income category stratified random effect meta-analysis 44](#_Toc177901152)

[Figure S 12: Age group stratified random effect meta-analysis 46](#_Toc177901153)

[Figure S 13: Sensitivity analysis using the leave-one-out meta-analysis method 47](#_Toc177901154)

**Appendix**

[Appendix 1: PRISMA checklist 48](#_Toc178031174)

[Appendix 2: Format for the quality assessment of the articles 50](#_Toc178031175)

Table S 1: Search strategy of databases

| PubMed | ("covid-19"[MeSH Terms] OR "sars-cov-2"[MeSH Terms] OR COVID-19 OR COVID19 OR covid2019 OR Coronavirus OR "Corona virus" OR coronavirus2019 OR "Novel coronavirus" OR "2019 novel coronavirus" OR "severe acute respiratory syndrome coronavirus 2" OR "SARS-CoV-2" OR SARS-2 OR 2019-nCoV OR ncov2019 OR "Coronavirus disease 2019" OR "COVID-19 infection" OR "COVID-19 patients" OR "COVID-19 survivors" OR "COVID-19 disease" OR coronavir* OR covid* OR ncov*) AND (“EQ-5D” OR “eq 5d” OR “eq5d” OR euroqol OR "euro qol" OR “euroQoL-5D” OR "euro QoL-5D" OR “EQ-5D-3L” OR “EQ-5D-5L” OR “EQ 5D-3L” OR “EQ 5D-5L” OR “EQ5D-3L” OR “EQ5D-5L” OR "EuroQol-5 Dimension" OR "EuroQol five dimension" OR "Euro QoL five dimension" OR "EuroQol 5 dimension" OR "Euro QoL 5 dimension")  Filter: English, publication years: 31/12/2019- 01/03/2025 | 710 |
| --- | --- | --- |
| Embase | ('covid 19'/exp OR 'covid 19' OR 'covid19'/exp OR covid19 OR covid2019 OR 'coronavirus'/exp OR coronavirus OR 'corona virus'/exp OR 'corona virus' OR coronavirus2019 OR 'novel coronavirus' OR '2019 novel coronavirus'/exp OR '2019 novel coronavirus' OR 'severe acute respiratory syndrome coronavirus 2'/exp OR 'severe acute respiratory syndrome coronavirus 2' OR 'sars-cov-2'/exp OR 'sars-cov-2' OR 'sars 2' OR '2019 ncov'/exp OR '2019 ncov' OR ncov2019 OR 'coronavirus disease 2019'/exp OR 'coronavirus disease 2019' OR 'covid-19 infection' OR 'covid-19 patients' OR 'covid-19 survivors' OR 'covid-19 disease' OR coronavir* OR covid* OR ncov*) AND ('eq-5d'/exp OR 'eq-5d' OR 'eq 5d'/exp OR 'eq 5d' OR 'eq5d'/exp OR 'eq5d' OR 'euroqol'/exp OR euroqol OR 'euro qol' OR 'euroqol-5d'/exp OR 'euroqol-5d' OR 'euro qol-5d' OR 'eq-5d-3l'/exp OR 'eq-5d-3l' OR 'eq-5d-5l'/exp OR 'eq-5d-5l' OR 'eq 5d-3l'/exp OR 'eq 5d-3l' OR 'eq 5d-5l'/exp OR 'eq 5d-5l' OR 'eq5d-3l' OR 'eq5d-5l' OR 'euroqol-5 dimension'/exp OR 'euroqol-5 dimension' OR 'euroqol five dimension'/exp OR 'euroqol five dimension' OR 'euro qol five dimension' OR 'euroqol 5 dimension'/exp OR 'euroqol 5 dimension' OR 'euro qol 5 dimension') AND [english]/lim : 31/12/2019- 01/03/2025 | 1383 |
| Scopus | TITLE-ABS-KEY ( ( covid-19 OR covid19 OR covid2019 OR coronavirus OR "Corona virus" OR coronavirus2019 OR "Novel coronavirus" OR "2019 novel coronavirus" OR "severe acute respiratory syndrome coronavirus 2" OR "SARS-CoV-2" OR sars-2 OR 2019-ncov OR ncov2019 OR "Coronavirus disease 2019" OR "COVID-19 infection" OR "COVID-19 patients" OR "COVID-19 survivors" OR "COVID-19 disease" OR coronavir* OR covid* OR ncov* ) AND ( eq-5d OR eq AND 5d OR eq5d OR euroqol OR "euro qol" OR euroqol-5d OR "euro QoL-5D" OR eq-5d-3l OR eq-5d-5l OR eq AND 5d-3l OR eq AND 5d-5l OR eq5d-3l OR eq5d-5l OR "EuroQol-5 Dimension" OR "EuroQol five dimension" OR "Euro QoL five dimension" OR "EuroQol 5 dimension" OR "Euro QoL 5 dimension" ) ) AND ( LIMIT-TO ( LANGUAGE , "English" ) ) AND ( LIMIT-TO ( PUBYEAR , 2020 ) OR LIMIT-TO ( PUBYEAR , 2021 ) OR LIMIT-TO ( PUBYEAR , 2022 ) ) PUBDATETXT ( March 2025 01] | 517 |
| Web of Science | TS= (COVID-19 OR COVID19 OR covid2019 OR Coronavirus OR "Corona virus" OR coronavirus2019 OR "Novel coronavirus" OR "2019 novel coronavirus" OR "severe acute respiratory syndrome coronavirus 2" OR "SARS-CoV-2" OR SARS-2 OR 2019-nCoV OR ncov2019 OR "Coronavirus disease 2019" OR "COVID-19 infection" OR "COVID-19 patients" OR "COVID-19 survivors" OR "COVID-19 disease" OR coronavir* OR covid* OR ncov*) AND (“EQ-5D” OR “eq 5d” OR “eq5d” OR euroqol OR "euro qol" OR “euroQoL-5D” OR "euro QoL-5D" OR “EQ-5D-3L” OR “EQ-5D-5L” OR “EQ 5D-3L” OR “EQ 5D-5L” OR “EQ5D-3L” OR “EQ5D-5L” OR "EuroQol-5 Dimension" OR "EuroQol five dimension" OR "Euro QoL five dimension" OR "EuroQol 5 dimension" OR "Euro QoL 5 dimension") Filter: English, publication years: 31/12/2019- 01/03/2025 | 648 |
| Cochrane Library | (COVID-19 OR COVID19 OR covid2019 OR Coronavirus OR "Corona virus" OR coronavirus2019 OR "Novel coronavirus" OR "2019 novel coronavirus" OR "severe acute respiratory syndrome coronavirus 2" OR "SARS-CoV-2" OR SARS-2 OR 2019-nCoV OR ncov2019 OR "Coronavirus disease 2019" OR "COVID-19 infection" OR "COVID-19 patients" OR "COVID-19 survivors" OR "COVID-19 disease" OR coronavir* OR covid* OR ncov*) AND (“EQ-5D” OR “eq 5d” OR “eq5d” OR euroqol OR "euro qol" OR “euroQoL-5D” OR "euro QoL-5D" OR “EQ-5D-3L” OR “EQ-5D-5L” OR “EQ 5D-3L” OR “EQ 5D-5L” OR “EQ5D-3L” OR “EQ5D-5L” OR "EuroQol-5 Dimension" OR "EuroQol five dimension" OR "Euro QoL five dimension" OR "EuroQol 5 dimension" OR "Euro QoL 5 dimension") in Title Abstract Keyword Filter: English, publication years: 31/12/2019- 01/03/2025 | 281 |

Table S 2: Basic characteristics of the included studies.

| **Author, publication year** | **Country** | **Study design** | **Sample size** | **Male (%)** | **Mean age(SD)** | **Time of assessment of QOL** | **Setting** | **Hospitalized (%)** | **DM (%)** | **HTN (%)** | **Asthma (%)** | **COPD (%)** | **CVD (%)** | **Kidney disease (%)** | **Malignancy (%)** | **No comorbidity (%)** | **Response rate (%)** | **NOS** |
| --- | --- | --- | --- | --- | --- | --- | --- | --- | --- | --- | --- | --- | --- | --- | --- | --- | --- | --- |
| Arab-Zozani et al., 2020 | Iran | Cross-sectional | 409 | 60.27 | 58.4(18.21) | 4 to 6 weeks | Patients discharged from hospital | 100 | 64.06 | 59.9 | - | - | - | - | - | - | 97.38 | 8 |
| Daher et al., 2020 | Germany | Cohort | 33 | 67 | 64(3) | 62 days | Patients discharged from hospital | 100 | 25 | 59 | 13 | 9 | 19 | 22 | - | - | 100 | 5 |
| Betschart et al., 2021 | Switzerland | Cohort | 43 | 70 | 60(14) | 12 months | Hospital | 100 | 23 | 51 | 5 | 7 | 35 | - | 16 | - | 79 | 4 |
| Meys et al., 2020 | Belgium | Cross-sectional | 210 | 12.4 | 45(11) | 79 days | Online social support group | 0 | - | - | - | - | - | - | - | 61.4 | 91 | 6 |
| Fernandes et al., 2021 | Portugal | Cross-sectional | 45 | 62.2 | - | 55 days | University hospital | 100 | - | 64 | - | - | - | 4 | - | - | 97.8 | 5 |
| Halpin et al., 2021 | UK | Cross-sectional | 100 | 54 | 66.8(14.9) | 48 days | Hospital discharge | 100 | 28 | 41 | 13 | 8 | 25 | 15 | 21 | - | 52.4 | 6 |
| Hodgson et al., 2021 | Australia | Cohort | 212 | 58.5 | 61(51-70)* | 6 months | 30 ICUs in six states | 100 | 32.5 | - | 13.9 | 8 | - | 6.9 | 5.9 | - | 77.3 | 5 |
| Huang et al, 2021 | China | Cohort | 1276 | 53 | 59(49–67)* | 6 and 12 months | Jin Yin-tan Hospital, Wuhan | 100 | 15 | 36 | - | 1 | 8 | 4 | 3 | - | 58 | 6 |
| Iqbal et al., 2021 | Pakistan | Cross-sectional | 158 | 44.9 | 40.1(12.42) | - | Karachi, laboratory | - | 9.5 | 13.3 | 10.1 | - | 7 | 1.3 | - | 60.1 | 79 | 8 |
| Johnsen et al., 2021 | Denmark | Cross-sectional | 57 | 49 | 51(13) | 3 months | Respiratory outpatient clinic | 60 | 9 | - | 26 | 4 | - | 4 | - | - | 48.7 | 4 |
| Kaso et al., 2021 | Ethiopia | Cross-sectional | 398 | 60 | 41.5(18.8) | - | Hospital-based | 100 | 17.1 | 10.3 | 8.3 | 7.5 | 5.8 | 1.8 | 2.8 | 55 | 94.3 | 9 |
| Kohlbrenner et al., 2021 | Switzerland | cohort | 58 | 65 | 60(49, 68)* | 3 months | Three participating centres | 100 | 17 | - | - | - | - | 28 | 14 | - | 69 | 6 |
| Kotwani P et al., 2021 | India | Cohort | 138 | 24.6 | 44.75(-11.78) | two weeks | Vadodara, Gujarat | 100 | - | - | - | - | - | - | - | - | - | 4 |
| Lerum et al., 2021 | Norway | Cohort | 103 | 52 | 59(49-72)* | 3 months | Six hospitals | 100 | 8 | 35 | - | - | - | - | - | - | 100 | 5 |
| Malinowska et al., 2021 | Poland | Cohort | 67 | 56.7 | 53.14(14) | 6 months | Single-center | 58.2 | 10.4 | - | - | - | - | 100 | - | - | 38.95 | 5 |
| Menges et al., 2021 | Switzerland | Cohort | 431 | 50.3 | 47(14.4) | 7.2 months | Community: contact tracing at the Department of Health of the Canton of Zurich | 19 | 5.6 | 15.8 | 9.3 | 1.9 | 2.8 | 1.4 | 3.7 | 65.7 | 97.5 | 5 |
| Monti et al., 2021 | Italy | Cohort | 39 | 90 | 56(10.5) | 61 days | Single-center teaching hospital | 100 | - | 49 | - | - | - | - | - | 46 | 93 | 5 |
| Och et al., 2021 | Poland | Cohort | 79 | 49.37 | 70(64–76.5)* | 3 and 6 months | 7th Navy Hospital in Gdańsk | 100 | 50.63 | 93.7 | - | - | - | 100 | - | - | 54.8 | 3 |
| Ordinola -varro et al., 2021 | Mexico | Cohort | 115 | 43 | 40(10) | 58 days | Specialty Hospital “Dr. Antonio Fraga Mouret” | 27 | 14 | 14 | - | - | - | - | - | - | 82 | 7 |
| Ozkeskin et al., 2021 | Turkey | Case-control study | 104 | 30.8 | 38.3(10.6) | 6 months | Ege university neurology department | - | - | - | - | - | - | - | - | - | - | 6 |
| Rousseau et al., 2021 | Belgium | Cohort | 32 | 72 | 62(49-68)* | - | Post-intensive care follow-up clinic | 100 | 62.5 | 53.1 | 12.5 | - | 31.2 | 6.2 | - | - | 80 | 5 |
| Shah AS et al 2021, | Canada | Cohort | 73 | 60 | 65(53-72)* | 3 and 6 months | Two academic hospitals | 100 | 26 | 37 | 4 | 5 | 10 | 8 | 11 | - | - | 4 |
| Shah et al., 2021 | Multicounty | Cross-sectional | 735 | 23.4 | 47.77(11.6) | 12.76 (6.104) weeks | Community | 20.1 | - | - | - | - | - | - | - | 69.1 | - | 8 |
| Tessitore et al., 2021 | Switzerland | Cohort | 165 | 62 | 58(50-69)* | 1 year | University Hospitals of Geneva | 100 | 16 | 35 | - | - | 21 | - | - | - | 24 | 4 |
| Todt et al., 2021 | Brazil | Cohort | 251 | 59.8 | 53.6(14.9) | 3 months | Hospital municipal dr. Moysés deutsch | 100 | 33.1 | 51.8 | 4.8 | 5.2 | 6.8 | 4 | 2.8 | - | 57 | 6 |
| Walle-Hansen et al., 2021 | Norway | Cohort | 106 | 57 | 74.3(11.4) | 6 months | Four general hospitals in South-Eastern Norway | 100 | 15 | 38 | - | 27 | 32 | - | - | - | 62 | 6 |
| Akova et al, 2022 | Turkey | Cross-sectional | 151 | 45 | 37.4(11.2) | - | outpatient clinic | 6.6 | 7.9 | 9.9 | - | - | 4.6 | - | - | 78.8 | 100 | 6 |
| Attauabi et al., 2021 | Denmark | Cohort | 516 | 49.8 | 46.4(15.9) | 5.1 MONTHS | All internal medicine and gastrointestinal departments in Denmark | 13.6 | 5.8 | 14.3 | 7.8 | 1.9 | 4.5 | 1.4 | 2.9 | 48.8 | 43 | 3 |
| Azizi et al., 2022 | Morocco | Case-control study | 1105 | 47.8 | 56.17(15.46) | 3 months | Patients discharged from hospital | 100 for cases | 27.2 | 28.4 | - | - | 9.1 | 31 | - | 48.3 | 83.5 | 7 |
| Barani et al., 2022 | India | Cross-sectional | 372 | 57.5 | 44.5(15.3) | Within 30 days | Community | 48.9 | 19.4 | 12.4 | 0.8 | - | 2.4 | 0.8 | 0.3 | 60.2 | 88.6 | 9 |
| Barreto et al., 2022 | Brazil | Cross-sectional | 1164 | 43.4 | 52.1(13.4) | 2.3 months (IQR = 1.6-3.7) | Outpatient clinic | 69.8 | 22.6 | 43.8 | 8.9 | 2.9 | 6.4 | - | - | 27.4 | 88.8 | 9 |
| Cuerda et al., 2022 | Spain | Cohort | 176 | 71.6 | 60.3(10.5) | 12.8 weeks | 16 public hospitals of the Community of Madrid | 100 | 18.8 | 46.3 | - | - | 18.2 | 9.1 | 9.1 | - | 94.6 | 5 |
| D’Ettorre et al., 2022 | Italy | Cross-sectional | 137 | 53.3 | - | 2 years | Infectious diseases ward | 100 | 9.5 | 25.5 | 8 | 6.6 | - | - | - | - | 69.2 | 8 |
| Oliveira et al., 2022 | Brazil | Cross-sectional | 439 | 50.3 | 58(47-67)* | Median 138 days (IQR 90-201) | Public hospital for infectious diseases | 100 | 26 | 44 | 2.5 | 4.8 | - | 2.5 | - | 25 | 59 | 5 |
| Demoule et al., 2022 | France | Cohort | 94 | 71 | 63(49-70)* | 2 and 12 months | Inpatient rehabilitation facility | 100 | 27 | 46 | 12 | 7 | - | 9 | - | - | 91 | 7 |
| Farhanah et al., 2022 | Indonesia | Cohort | 104 | 52.9 | 48.96(15.97) | 3 months | Dr. Kariadi Hospital Semarang | 100 | 28.8 | 31.7 | 4.8 | 4.8 | 7.6 | 3.8 | 6.7 | 30.8 | 92.9 | 5 |
| Fontes etal., 2022 | Portugal | Cross-sectional | 99 | 63.6 | 63(12) | 53 ± 21 days | Department of Intensive Care Medicine of São João University Hospital Center | 100 | - | - | - | - | - | - | - | - | 89.2 | 5 |
| Haberland et al., 2022 | Germany | Cohort | 412 | 41.8 | 47(15.2) | 203.5 days (mean) | Community | 2.4 | 0.5 | 13.1 | 8.9 | 1.5 | - | - | - | - | 23.4 | 4 |
| Han et al., 2022 | United States | Cohort | 213 | 34.3 | 45(12) | 6-11 months | Eight academic medical centers | - | - | - | - | - | - | - | - | - | 21 | 4 |
| Hegde et al., 2022 | India | Cohort | 123 | 58.5 | 45.28(17.76) | 6 months | Admitted to the hospital and recovered patients | 100 | - | - | - | - | - | - | - | - | - | 6 |
| Heubner et al., 2022 | Germany | Cohort | 184 | 72.8 | - | 6 months | Intensive care unit | - | 42.9 | 71.2 | - | 7.1 | 24.5 | 15.2 | - | 3 | 72.4 | 4 |
| Huynh et al, 2022 | Viet-m | Cross-sectional | 325 | 38.2 | - | - | Two hospitals in Ho Chi Minh City | - | 11.1 | 8 | - | - | - | 2.5 | - | 67.4 | 96.2 | 9 |
| Kalyani et al. 2022 | India | Cross-sectional | 86 | 70.93 | - | 3 months | Tertiary care hospital | 100 | 10.46 | 25.58 | 15.11 | - | - | 6.97 | - | - | - | 4 |
| Kaso et al., 2022 | Ethiopia | Cross-sectional | 493 | 64.9 | - | - | Three treatment centers in Ethiopia | 100 | 13 | 7.5 | 7.9 | 7.5 | 4.5 | 3.7 | 3.7 | 69.4 | 91 | 9 |
| Koullias et al., 2022 | Greece | Cohort | 151 | 51.7 | 46.22(15.74) | At least 6 months | General Hospital of Athens | 24.5 | - | - | - | - | - | - | - | - | - | 7 |
| Lim et al., 2022 | Canada | Cohort | 84 | 43 | 46.5(11.3) | 3 months and 12 months | Outpatient | - | 25 | 25 | 17 | - | - | - | - | - | - | 6 |
| Luong et al., 2022 | USA | Cohort | 111 | 44.1 | 43(15.4) | 4 months | Academic medical center | - | - | - | - | - | - | - | - | - | - | 3 |
| Martins et al., 2022 | Portugal | Cohort | 56 | 68 | 65(14.6) | - | Single-center, Intensive Care Medicine Department | 100 | - | - | - | - | - | - | - | - | 81 | 5 |
| Moens et al., 2022 | Belgium | Cross-sectional | 547 | 13.7 | 46.6(11.5) | 287 days (150) | Community | - | - | - | - | - | - | - | - | - | - | 5 |
| Morrow et al., 2022 | UK | Cohort | 159 | 56.6 | 54.5(11.9) | 28-60 days post-discharge | Multi-center | 90 | - | - | - | - | - | - | - | - | - | 6 |
| Nakshbandi et al., 2022 | The Netherlands | Cohort | 133 | 70.1 | 60(10.42) | 6 MONTHS | Three hospitals | 100 | 19.7 | - | 11.1 | 6.8 | 42.7 | - | 2.6 | - | 87.9 | 6 |
| Ojeda et al., 2022 | Spain | Cohort | 65 | 73.8 | 65(57-70)* | 1 month after hospital discharge | Hospital Clinic of Barcelona | 100 | 29.2 | 53.8 | 3.1 | 9.2 | 9.2 | 7.7 | 4.6 | 10.8 | 31.9 | 6 |
| Pacho-Hernández et al., 2022 | Spain | Cross-sectional | 146 | 46.6 | 57.5(12) | 18.8 months | Urban hospitals | 100 | - | - | - | - | - | - | - | - | - | 6 |
| Pan et al., 2022 | Chi- | Cohort | 45 | 51.1 | 51(42-56)* | 1 and 2 years | Taizhou Hospital in Zhejiang Province | 100 | - | - | - | - | - | - | - | 56 | 61.7 | 4 |
| Said et al., 2022 | USA | Cross-sectional | 286 | 18.18 | 37.1(13.08) | - | Tertiary medical center and social media support forum | 3.15 | 2.8 | 10.49 | - | - | - | - | 0.7 | - | - | 8 |
| Schallner et al., 2022 | Germany | Cohort | 49 | 78 | 58.1(10.5) | 6 months | Tertiary care-level ICU and acute respiratory distress syndrome/ECMO centre | 100 | - | - | - | - | - | - | - | - | 45 | 4 |
| Soh and Cho, 2022 | South Korea | Cohort | 147 | 53.7 | 51(43.0–61.0)* | 3 months after discharge | Seongnam Coronavirus Treatment Center | - | 10.9 | 27.9 | 2.7 | - | 0.7 | 0.7 | 7.5 | 45.6 | 73.1 | 6 |
| Tabacof et al., 2022 | USA | Cross-sectional | 156 | 31 | 44(13.5) | Median 351 days (range 82-457 days) | Mount Sinai’s Post-acute COVID-19 syndrome clinic | 11 | - | 7 | 20 | - | - | - | 20 | - | 48 | 6 |
| Tak, 2023 | Multi-country study | Cross-sectional | 82 | 36.6 | 37(15.2) | Mean 13 months (SD 6.5) | Online | 3.7 | - | - | - | - | - | - | - | - | - | 7 |
| Tarazo- et al., 2022 | France | Cohort | 177 | 38.9 | 44.5(14.7) | 14 to 20 months | Four COVID outpatient centres | 0 | 4.5 | - | - | - | - | - | - | - | 43 | 8 |
| Tsuzuki et al., 2022 | Japan | Cross-sectional | 457 | 49.5 | 47(10.6) | At least 56 days | Outpatient service of National Center for Global Health  and Medicine | 56.5 | 6.1 | 14.4 | - | 0.7 | - | - | 1.3 | - | 86.9 | 9 |
| Umbrello et al., 2022 | Italy | Cohort | 79 | 84 | 63(57-71)* | 6 months after ICU discharge | Two referral hospitals in the northeast Italian region Trentino-Alto Adige | - | 11 | 46 | 13 | 13 | 8 | - | 5 | 48 | 85 | 7 |
| Vejen et al., 2022 | Denmark | Cohort | 128 | 58 | - | 4-5 months after discharge | Respiratory outpatient clinic | 100 | 28 | 48 | 9 | 7 | - | - | - | - | 80 | 4 |
| Weihe et al., 2022 | Denmark | Cohort | 326 | 68 | - | 6 and 12 months for follow-up | 29 Danish ICUs | - | 19 | 47 | - | 15 | 12 | 12 | 6 | 0.34 | 51 | 4 |
| Wimmer et al., 2022 | Germany | Cohort | 61 | 73.8 | 61.9(12.9) | 120.4 days (36.9) at discharge | Inpatient neuro-rehabilitation | 100 | 24.6 | 42.6 | - | - | - | 32.8 | - | - | 53.8 | 4 |
| Wu D, et al. 2022 | China | Cross-sectional | 10 | 30 | 62.5(15.6) | After discharge was 5.8±0.6 months | Wuhan union hospital | 100 | - | - | - | - | - | - | - | - | 80 | 5 |
| Zhang et al., 2022 | China | Cross-sectional | 255 | 50.98 | 43.78(16.08) | One year | Shandong province | 100 | - | - | - | - | - | - | - | - | - | 8 |
| Fietsam et al., 2023 | USA | Cross-sectional | 49 | 37.5 | 34.6(13.8) | 6.7 (4.4) months | University of Iowa | - | - | - | - | - | - | - | - | - | - | 4 |
| Hoque et al., 2023 | Bangladesh | Cross-sectional | 563 | 55.95 | 51.18(13.49) | One month after discharge | Post-covid clinic of Dhaka Medical College Hospital | 100 | 40.5 | 30.2 | - | - | - | - | - | 38.9 | 94.8 | 9 |
| Huarcaya-Victoria et al., 2022 | Peru | Cohort | 119 | 53.8 | 55(14) | 3 and 12 months after hospital discharge_ | Hospital Nacional Guillermo Almenara Irigoyen | 100 | - | - | - | - | - | - | - | - | 37.4 | 6 |
| Iribarren-Diarasarri et al., 2022 | Spain | Cohort | 143 | 68.5 | 61.12(16.21) | one month after discharge | Two multipurpose critical care units of the Araba University Hospital | 100 | - | - | - | - | - | - | - | - | 77.3 | 7 |
| Román-Montes et al., 2023 | Mexico | Cross-sectional | 246 | 54.87 | 52.5(41–64)* | 150 days | National Institute of Medical Sciences and Nutrition Salvador Zubirán | 100 | 23 | 33 | 3 | 2 | 4 | 6 | 3.2 | - | 78.8 | 8 |
| Rosa et al., 2023 | Brazil | Cohort | 1508 | 60.8 | - | 12 months | 84 sites in Brazil | 100 | 24.2 | 45.2 | 5.3 | 2.7 | 2.5 | 2.1 | 2.9 | - | 85.4 | 6 |
| Rousseau et al., 2023 | Belgium | Cohort | 143 | 62.9 | 62.5(13.6) | 3 months after discharge | Post-ICU follow-up clinic | 100 | 28 | 40.6 | - | - | 19.6 | 7.7 | - | - | 59.6 | 4 |
| Sánchez-García et al., 2023 | Spain | Cohort | 104 | 77.9 | 56.7(13.8) | one month after hospital discharge | Outpatient clinic | 100 | 27.9 | 48.1 | 8.7 | 2.9 | 10.6 | 1.9 | - | - | 28.8 | 5 |
| Shah et al., 2023 | India | Cohort | 388 | 62.6 | 48(36-59)* | 1 and 3 months | Tertiary care centre | 100 | 29.38 | 35 | 3.35 | - | 3.09 | - | 0.77 | - | 81 | 6 |
| Wong et al,.2023 | Canada | Cohort | 1,344 | 42 | 51(15) | 3 and 6 months | Post-COVID recovery clinics | 42 | 18 | 29 | 20 | 7 | 12 | - | 3 | - | - | 6 |
| Taboada et al., 2020 | Spain | Cohort | 91 | 66 | 74.3(9.1) | 6 months | Seven hospitals in north western Spain | 100 | 24 | 55 | 4 | 8 | 10 | 7 | 5 | 23 | 62 | 6 |
| Ferrarello et al., 2023 | Italy | Cohort | 44 | 41 | 67(15.9) | 6 months | Outpatient functional rehabilitation clinic | 86 | 18 | 48 | - | - | - | - | - | 12 | - | 6 |
| Slotegraaf et al, 2023 | Netherlands | Cohort | 1451 | 36.2 | 49(13) | 3 and 6 months | Allied health care in Dutch primary care | 22.8 | - | - | - | - | 14.5 | - | - | 57.6 | 63.5 | 7 |
| Zupanc et al., 2023 | Slovenia | Cohort | 157 | 68 | 64(9) | - | Inpatient rehabilitation | 100 | - | - | - | - | - | - | - | - | 100 | 4 |
| Giurgi-Oncu et al., 2021 | Romania | Cross-sectional | 143 | 45.45 | 44.06(9.12) | 4 to 12 weeks | Outpatient clinic | 44.75 | - | - | - | - | - | - | - | - | 91.1 | 9 |
| Cavalleri et al., 2022 | Belgium | Cohort | 220 | 66.8 | 65(11) | 1 year | Two public hospitals | 100 | 38.6 | 65.5 | 8.6 | 10.9 | 52.7 | 13.2 | - | - | 60.4 | 4 |
| Sandmann et al., 2021 | England | Cohort | 1199 | 38.5 | 41.1(16.9) | Up to 6 months | Community | - | 3.8 | 9.7 | 13.1 | - | - | - | - | 72.5 | 36.5 | 7 |
| Carenzo et al., 2021 | Italy | Cohort | 47 | 79 | 59(10) | 2 and 6 months | Follow-up clinic | 100 | - | - | - | - | - | - | - | - | 96 | 5 |
| Morelli et al., 2022 | USA | Cross-sectional | 92 | 49 | 59.9(11.6) | 1 month | Outpatient academic medical clinic | 61 | - | - | - | - | - | - | - | - | - | 4 |
| Evans et al., 2022 | UK | Cohort | 2320 | 61 | 58(12.6) | 5 months | 83 NHS hospitals across the UK | 100 | 28·9 | 51·9 | 14·6 | 7·5 | 15·1 | 10·4 | 8·0 | 27·7 | 32·7 | 6 |
| Brus et al., 2023 | Netherlands | Cross-sectional | 10194 | 23.9 | 49 (16) | ≥3 | Online survey via a national support organization | 8.3 | - | - | - | - | - | - | 52.7 | 52.7 | 57.5 | 6 |
| Sawano et al., 2025 | multicountry | Cross-sectional | 441 | 26 | 46 (38-57) | 14.9 | Online digital research initiative (Yale LISTEN study) | - | 2 | 3 | - | 5 | - | - | - | - | - | 3 |
| Scott et al., 2023 | multicountry | Cohort | 240 | 41.7 | 49.6 (12.9) | ≥3 | Web-based general population survey | - | - | - | - | - | - | - | 40 | 0.4 | 0.25 | 7 |
| Khoja et al, 2024 | UK | Cross-sectional | 30 | 36.7 | 46.8 (11.1) | 17.1 | Community Rehabilitation Service | 10 | 6.7 | 10 | 10 | 0 | - | - | 40 | 0.4 | - | 4 |
| Cataldo et al, 2024 | Argentina | Cross-sectional | 109 | 27.5 | 48.4 (8) | 29.9 | Community-based | 16.5 | 30.3 | 7.3 | - | - | - | - | - | - | - | 5 |
| Wemhöner et al., 2025 | Germany | Cohort | 51 | 49 | 80.2 (6.4) | 12 | Single academic center | 100 | - | - | - | - | - | - | - | - | 26.4 | 5 |
| Engel et al., 2025 | Germany | Cohort | 22 | 69.6 | 57 (52-65) | 3 and 6 | Single academic center | 100 | - | 26.1 | 50.9 | - | - | - | - | - | 43 | 5 |
| Malesevic et al., 2023 | Switzerland | Cross-sectional | 112 | 23.2 | 43 (32.0-52.5) | 5.1 | Outpatient clinic | - | 13.4 | - | - | - | - | - | 75 | 75 | 37.2 | 5 |
| Ding et al., 2024 | China | Cross-sectional | 648 | 56.6 | 35.6 (15) | - | Online survey | - | - | - | 12.5 | - | - | - | 87.3 | 87.5 | - | 5 |
| Crescioli et al., 2024 | multicountry | Cohort | 726 | 68.2 | 60 (51-70) | 12 | 11 ICUs | 100 | 7.2 | 7.7 | 10.6 | - | - | - | - | - | 88.4 | 6 |
| Egger et al., 2024 | Germany | Cohort | 97 | 69.1 | 61.1 (12.1) | 12 | Single neurorehabilitation center | 100 | - | - | - | - | - | - | - | - | 76.3 | 5 |
| Neelima et al., 2023 | India | Cross-sectional | 107 | 66.4 | 55.24 (9.94) | ~8 | Single tertiary care center | 100 | 6.5 | - | - | - | - | - | 35.5 | 35.5 | - | 6 |
| Leavy et al., 2024 | United Kingdom | Cohort | 1888 | 64.4 | 58.6 (-) | 12 | Multicenter (36 UK hospitals) | 100 | 14.1 | 9.1 | 49 | 8.8 | 8.9 | - | 29.8 | 29.8 | 64.9 | 8 |
| Berentschot J. C. et al., 2024 | Netherlands | Cohort | 650 | 69 | 59.7 (11.4) | 3, 6, 12, 24 | Multicentre (7 hospitals, 3 rehab centers) | 100 | - | - | 40 | 9 | 11 | - | 17 | 17 | 78.3 | 7 |
| Zhao X, et al., 2024 | China | Cohort | 285 | 67 | 51.96 (15.89) | 3, 6, 12 | Single-center (Hospital) | - | - | - | 17.5 | 100 | - | - | 77.9 | - | 77.9 | 5 |
| Colleran R, et al., 2023 | Ireland | Cohort | 100 | 36 | 45.2 (-) | 6, 12 | Community medicine | 0 | - | - | - | - | - | - | 77 | - | 72 | 4 |
| Visser et al., 2024 | Netherlands | Cohort | 465 | 67.3 | 60.3 (12.2) | 3 | 4 academic hospitals | 100 | 21.4 | 37.6 | 6.6 | 9.3 | 8.7 | 22.8 | - | 22.8 | - | 8 |
| Neumann et al., 2025 | Germany | Cohort | 3475 | 44 | 44 (32-57) | 9 and 26 | Population-based (recruited via public health authorities) | - | 1.3 | - | - | 1.3 | - | - | 72.2 | - | 72.2 | 7 |
| Appel et al., 2024 | Germany | Cohort | 603 | 60.6 | 54 (16.1) | 3 and 12 | Multi-sectoral (university hospitals, other hospitals, primary care) | 82 | - | 46.1 | 10.6 | 17.1 | - | 29.4 | 68.5 | 29.4 | 68.5 | 5 |
| Deesomchok et al., 2023 | Thailand | Cohort | 63 | 50.8 | 41.1 (14.8) | 1, 3, 6, 9, 12 | Single university hospital outpatient clinic | 100 | - | 30.2 | - | - | - | 68.3 | 77.8 | 68.3 | 77.8 | 6 |
| Kato et al., 2025 | Japan | case-control | 609 | 100 | 56 (48-63) | 12 and 24 | 20 hospitals in Japan | 100 | - | - | - | - | - | - | - | - | - | 7 |
| Rego de Figueiredo et al., 2023 | Portugal | Cross-sectional | 125 | 49 | 70 (13) | 3, 6, 12 | Single-center hospital | 100 | - | 22 | - | - | - | - | - | - | 0.3 | 7 |
| Gorsler et al., 2024 | Germany | Cohort | 30 | 66.7 | 64 (57-68) | ~1.2 months (duration of stay) | Single neurological rehabilitation clinic | 100 | 16.7 | 16.7 | 23.3 | 6.7 | 6.7 | - | 37 | - | 0.37 | 4 |
| Naik et al., 2025 | Canada | Cross-sectional | 1135 | 51.3 | 41 (29-50) | 28 | Population-based (Province of British Columbia) | 2.8 | - | - | - | - | - | - | 81.3 | 0.813 | 0.39 | 8 |
| Sun X et al., 2024 | US | Cohort | 328 | 26.2 | 42 (14.5) | 1, 3, 6 | Community (recruited from pharmacy test sites) | 0 | 9.2 | - | 12.5 | - | - | - | 73.5 | 0.735 | 0.49 | 4 |
| Hatakeyama et al., 2025 | Japan | Cohort | 334 | 79.6 | 67 (58-74) | 5.5, 12.5, 18.5, and 24.5 | Multicenter (32 ICUs) | 100 | - | 9 | 11.1 | 2.7 | 6.6 | - | 32.6 | 32.6 | 79.9 | 7 |
| Soare et al., 2024 | United Kingdom | Cross-sectional | 406 | 45.2 | 48 (15.3) | 12 | Online recruitment | 13.1 | - | - | - | - | - | - | 43 | 43 | - | 6 |
| Rover et al., 2024 | Brazil | Cohort | 1067 | 30.8 | 39 (30.8-50.0) | 3 | Multicenter (14 centers) | 0 | - | - | - | - | - | - | 84.4 | 84.4 | 88.4 | 8 |
| Janols et al., 2024 | Sweden | Cross-sectional | 177 | 44.6 | 52 (11.5) | >3 | Single-center (University Hospital) | - | - | 27 | 7 | - | 3 | - | - | - | 51.9 | 4 |
| Guaraldi et al., 2023 | Italy | Cross-sectional | 232 | 60.8 | 58 (50.0-67.0) | ≥3 | Single-center (PACS Clinic) | 100 | - | - | - | - | - | - | - | - | - | 7 |
| Kwon et al, 2024 | UK | Cross-sectional | 366 | 32.2 | 48.3 (11.7) | 14.8 | Specialist clinics | 10.9 | - | - | - | - | - | - | - | - | 51.3 | 7 |
| Smith et al, 2023 | Belgium | Cohort | 5727 | 43.9 | - (-) | 3 | Population-based | - | - | - | - | - | - | - | 92.6 | 92.6 (weighted) | 79 | 8 |
| Tak, 2023 | multicountry | Cross-sectional | 82 | 36.6 | 37 (-) | 13 | Online survey (Social media) | 100 | - | - | - | - | - | - | - | - | - | 6 |
| Qorolli et al., 2023 | Kosovo | Cross-sectional | 39 | 56.4 | 50.1 (12.2) | 0.5 | Single university clinical center | 100 | - | - | - | - | - | - | - | - | 36.4 | 4 |
| Tiels et al., 2025 | Netherlands | Cohort | 87 | 71.3 | 61 (-) | 3, 12, 24 | Single university medical center | 100 | - | 13.8 | 8 | - | 6.9 | - | - | - | 43.9 | 6 |
| Hansen KS, et al., 2023 | Denmark | Cohort | 279 | 25.5 | 49 (39-57) | 6.5 | Multicenter | 0 | 14.3 | 0.7 | - | - | - | - | - | - | - | 7 |
| Mastrorosa I, et al., 2023 | Italy | Cross-sectional | 572 | 53 | 55 (47-62) | 4.8 | Single-center | - | - | - | - | - | - | - | - | 62.4 | - | 8 |
| Elneima O, et al., 2024 | United Kingdom | Cohort | 615 | 51.5 | 58.7 (12.9) | 12 | Multicenter (National) | 54 | - | - | - | - | - | - | 62.4 | 22.4 | 77.9 | 8 |
| Sun et al., 2023 | United States | Cross-sectional | 676 | 26.8 | - (-) | 0 | National test sites (CVS Health) | 100 | 3.39 | 3.39 | - | 0.5 | - | - | 81 | - | 1.7 | 7 |
| Fernández-de-las-Peñas et al., 2023 | Spain | Cross-sectional | 77 | 62.4 | 60 (11.5) | 6 | Single urban hospital | 0 | - | - | - | - | - | - | 1.7 | - | - | 4 |
| Mercier et al., 2023 | Canada | Cohort | 413 | 33.2 | 48.3 (16.1) | 3, 6, 12, 18, 24 | Single site of a multicenter biobank | 100 | 13 | 6.5 | - | - | - | - | - | - | 48 | 8 |
| Tsuruoka et al., 2025 | Vietnam | Cohort | 342 | 38.3 | 45 (31-60) | 6 and 16 | Three major hospitals | 100 | 16.2 | 16.2 | 9.1 | - | - | - | 58.9 | - | 54.4 | 5 |
| van Tol et al., 2024 | multicountry | Cohort | 723 | 52.4 | 75.49 (9.91) | 0 to 6 months | 59 Geriatric Rehabilitation (GR) facilities | 1.8 | 0 | 0.3 | 6.8 | 6.2 | 0.9 | 0.3 | - | - | - | 7 |
| Carlile et al., 2024 | United Kingdom (England) | Cohort | 6070 | 33.8 | 53 (43-62) | 0 to 3 months | Primary care (via OpenSAFELY platform) | 90.7 | - | - | - | - | - | - | - | 56.7 | - | 5 |
| Carrera et al., 2025 | Argentina | Cohort | 51 | 66.6 | 53.43 (10.94) | 0 to 12 months | Single-center ICU follow-up clinic | 3 | - | - | - | - | - | - | 56.7 | - | 87.9 | 4 |
| Elumalai et al., 2023 | India | Cross-sectional | 1047 | 68 | 38 (29-51) | >5 | Siddha COVID Care Centres | 100 | - | - | - | - | - | - | 87.9 | 75 | 52.4 | 8 |
| Di Fusco et al., 2023 | US | Cohort | 328 | 26.2 | 42 (14.5) | 1, 3, 6 | Online surveys (CVS Health test sites) | 100 | - | 1.9 | - | - | - | 75 | 52.4 | 73.5 | 79.2 | 7 |
| Kuodi et al., 2023 | Israel | Cross-sectional | 951 | 34.3 | 46 (14.7) | 18 | Multicenter (3 hospitals) | - | 9.2 | - | - | - | - | - | 73.5 | - | 7.3 | 7 |
| Godfrey et al., 2025 | United Kingdom | Cohort | 31 | 35 | 47 (11) | 0-3 | Community rehabilitation service | 14.3 | 3.6 | 0.9 | - | 0.6 | - | - | - | - | 66 | 5 |
| Lomholt-Welch et al., 2023 | United Kingdom | Cohort | 152 | 57 | 54.3 (11.8) | 2 | Multicenter (3 hospitals) | - | 16 | - | 3 | - | 3 | - | - | - | 20.4 | 4 |
| Atchison et al., 2023 | England | Cross-sectional | 3222 | 41.6 | - (-) | 13 | Community | 100 | - | - | 45 | 5 | - | - | - | 64.5 | 34.6 | 8 |
| Walker et al., 2023 | UK (England and Wales) | Cross-sectional | 3754 | 28.2 | 48 (12) | ≥3 | Post-COVID-19 clinics (using a digital health interface) | 90.3 | - | - | - | - | - | - | - | - | - | 7 |
| Schröder et al., 2024 | Germany | Cross-sectional | 3188 | 21 | 42.8 (12.2) | ≥1 (mean ~12.3) | Online survey | 100 | 3 | 2 | 4.2 | 4.8 | 2.5 | 1 | - | - | 0.561 | 7 |
| Duwel et al., 2023 | Aruba | Cross-sectional | 222 | 53 | 58.1 (12.8) | 3, 6, 12, 18 | Primary health care facility | - | - | 17.8 | - | 3.7 | - | - | 56.1 | 17 | 34.5 | 8 |
| Pietruszka-Wałęka et al., 2024 | Poland | Cohort | 46 | 52 | 63 (53-69) | 3 and 9 | Tertiary hospital | 100 | - | - | - | - | - | 17 | 34.5 | - | 100 | 6 |
| Samuelsson et al., 2025 | Sweden | Cohort | 105 | 76 | 58 (51-66) | 12 | Multi-ICU | 100 | - | - | 49 | 27.4 | - | - | 29.8 | - | 0.58 | 8 |
| Seeley et al., 2025 | Australia | Cohort | 99 | 18.2 | 37 (15) | ~10.7 | Specialist cardiology clinic and social media | - | - | - | - | - | - | - | - | - | 48.5 ( | 4 |
| Agergaard et al., 2023 | Denmark | Cohort | 806 | 30 | 48 (37-56) | 7, 8, 10, 12, 18 | Post-COVID Clinic (tertiary hospital) | 3 | 15.2 | - | - | - | - | - | - | - | 86 | 5 |
| Amedewonu et al., 2024 | Ghana | Cross-sectional | 150 | 45.3 | 43.3 (14.8) | ≥ 1 | Infectious disease centre review clinic | 2.9 | - | - | - | - | - | - | 56.7 | 58 | - | 7 |
| D'Souza et al., 2024 | Australia | Cohort | 143 | 32 | 42 (32.00–51.00) | 12 | Outpatient rehabilitation service | - | 65.1 | 12.7 | - | 1.6 | - | - | 58 | - | 27 | 4 |
| Carenzo et al., 2024 | Italy | Cohort | 105 | 78 | 60 (10) | 18 | ICU follow-up clinic | - | - | - | - | - | - | - | 27 | - | 71.9 | 4 |
| Mccarthy et al., 2024 | Republic of Ireland | Cohort | 20 | 50 | 74.4 (11.8) | 6 | Two acute hospital sites | 100 | - | - | - | - | - | - | 71.9 | 0 | 66.7 | 5 |
| Malesevic et al., 2023 | Switzerland | Cohort | 69 | 20 | 44.2 (11.9) | ~11 | University Hospital | 100 | - | - | - | - | - | 0 | 66.7 | 79.7 | 61.6 | 5 |
| Dennis et al., 2023 | United Kingdom | Cohort | 331 | 27 | 47 (11) | 12 | Non-acute healthcare settings | 0 | 13 | - | 20.3 | - | - | - | 79.7 | - | 85.3 | 6 |
| Cannata et al., 2023 | Italy | Cohort | 110 | 65.5 | 65 (57-72) | 7 | Academic tertiary hospital | 100 | - | - | - | - | - | - | - | - | 94 | 7 |
| Wang J et al., 2024 | UK | Cohort | 3523 | 28.3 | 47.3 (12.2) | 6 | 35 specialised long COVID clinics | 100 | - | 3.6 | - | 11.8 | 12.7 | - | - | - | - | 5 |
| Wangnamthip S et al., 2024 | Thailand | Cross-sectional | 1019 | 41.1 | 46.5 (17.2) | 6 | Single-center hospital and home monitoring | 100 | 5.8 | - | - | - | - | - | - | 55.2 | 68.4 | 7 |
| Caamano E et al., 2024 | Spain | Cohort | 101 | 66 | 59 (46-66) | 12 | ICU of a tertiary care hospital | 64.9 | - | - | - | - | - | - | 55.2 | 24.8 | 87.1 | 6 |
| Sinaga et al., 2023 | Indonesia | Cross-sectional | 100 | 14 | - (-) | >1 | Single hospital | - | - | - | - | - | - | - | - | 0.58 | - | 5 |
| Tabacof et al., 2023 | US | Cohort | 99 | 33 | 45 (-) | 7.2 | Hospital Clinic | 100 | - | - | - | - | - | - | - | - | 0.488 | 6 |
| Thanh HN et al., 2024 | Vietnam | Cross-sectional | 394 | 36.3 | 29.46 (12.17) | 2.1 | Community | - | - | - | - | - | - | - | - | 0.817 | 0.923 | 9 |
| Cijs et al., 2023 | Netherlands | Cohort | 183 | 68.9 | 57.6 (13.3) | 3 | Hospital (ICU aftercare clinic) | 6.8 | - | - | - | - | - | - | 81.7 | - | - | 8 |
| Domazet Bugarin et al., 2023 | Croatia | Cohort | 113 | 74 | 61.2 (-) | 4 | Tertiary hospital center | 100 | 36 | - | 12 | - | 13 | - | 33 | 33 | 87.6 | 7 |
| Cázares-Lara et al., 2024 | México | Cross-sectional | 179 | 36 | 33 (-) | 7.3 (Median) | Tertiary medical center | 14 | 12 | - | - | - | - | - | 79 | 79 | 29.9 | 7 |
| Pavithra et al., 2023 | India | Cross-sectional | 100 | 56 | - (-) | < 6 | Hospital-based (OPD) | 44 | 16 | 5 | 4 | 2 | 2 | - | - | - | - | 3 |
| Bolgeo et al., 2024 | Italy | Cohort | 199 | 57.8 | 53.14 (16.56) | 4 and 8 | Multicenter (3 hospitals) | 100 | 52.3 | - | 10.6 | 26.6 | 2 | 4 | - | 0.251 | 72.9% | 7 |
| Salem et al., 2023 | Egypt | Cross-sectional | 477 | 45.5 | - (-) | ≥0.75 | Single teaching hospital | 18.4 | - | 4 | 4.4 | - | - | - | - | - | - | 7 |
| Honda et al., 2025 | Japan | Cross-sectional | 19784 | 48.5 | 49 (34-67) | ≥2 | Nationwide online survey | - | 13.3 | 3 | 5.4 | 4.3 | 8.7 | - | - | - | - | 7 |
| Gharibzadeh et al., 2024 | United Kingdom | Cohort | 2545 | 61.1 | 58 (12.6) | 12 | 83 National Health Service (NHS) hospitals | 100 | 34.5 | - | 22.8 | - | 4.2 | - | - | 0.469 | - | 8 |
| Berentschot et al., 2024 | The Netherlands | Cohort | 502 | 69.7 | 60 (-) | 24 | 7 hospitals and 3 rehabilitation centers | 100 | - | 24.2 | 24.2 | 37.8 | - | - | - | - | 0.772 | 6 |
| Macedo Junior et al., 2024 | Brazil | Cross-sectional | 109 | 32.1 | 44 (-) | 13 | Quaternary hospital | 5.5 | 29.3 | 2.2 | - | - | - | - | 0 | 0 | 0.258 | 7 |
| Álvarez-Hernández et al., 2023 | Spain | Cohort | 199 | 70.4 | 60.7 (10.1) | 12 | 16 public hospitals in Madrid | 100 | - | 27.1 | 19.1 | 9 | 10.1 | - | - | - | - | 5 |
| Prata TA, et al., 2024 | Brazil | Cross-sectional | 189 | 49.2 | 59.6 (13.4) | 12 | Multicenter (3 public referral hospitals) | 100 | 11.1 | 6.7 | 12.4 | 5 | - | - | 11.2 | 11.2 | 41.6 | 5 |
| Alanazi MQ, et al., 2023 | Saudi Arabia | Cohort | 389 | 33.4 | 34.9 (8.4) | 0.5 | Single-center (tertiary care clinic) | 0 | 11.5 | 15.9 | - | - | - | - | 44.3 | 44.3 | 65.9 | 5 |
| Moisoglou et al., 2024 | Greece | Cross-sectional | 122 | 27 | 44.8 (11.5) | 11.6 | Online (recruited from a patient society Facebook page) | 20.5 | - | - | - | - | - | - | - | - | - | 7 |
| Ramos et al., 2024 | Brazil | Cohort | 186 | 61 | 67 (-) | 6 | Post-acute care facility (PACF) | 100 | - | - | - | - | - | - | - | - | 98.4 | 7 |
| Gursoy et al., 2023 | Türkiye | Cross-sectional | 145 | 56.6 | 62.39 (14.71) | 3-12 (mean 11.7) | Tertiary hospital outpatient clinic | 100 | - | - | - | - | - | - | - | - | - | 5 |
| Wang R et al., 2023 | China | Cross-sectional | 1239 | 39 | 26.02 (10.27) | 0 | Online survey (WeChat) | 0 | - | - | - | - | - | - | 86.8 | 86.8 | 95.4 | 8 |
| Demirhan et al., 2023 | Turkey | Cross-sectional | 392 | 50 | 51.27 (11.13) | 3 | Tertiary hospital (phone survey) | 100 | - | - | 15.3 | 5.6 | 5.9 | - | 33.2 | 33.2 | 43.8 | 5 |
| Firouzabadi et al, 2024 | Iran | Cross-sectional | 194 | 51 | 50.1 (15.8) | 6 | Hospital and clinics | 58.2 | 3.6 | - | 11.9 | - | 2.1 | - | - | - | 67.1 | 7 |
| Sun et al, 2024 | China | Cross-sectional | 307 | 30.62 | - (-) | 2 | Community | 0 | - | - | - | - | - | - | 85.34 | 85.34 | 97.8 | 7 |
| Galanis et al., 2023 | Greece | Cross-sectional | 122 | 27 | 44.8 (11.5) | 11.6 (mean duration of symptoms) | Online (convenience sample from patient society) | 20.5 | - | - | - | - | - | - | - | - | - | 7 |
| Dodd et al., 2024 | Australia | Cohort | 144 | 58.33 | 46.9 (14.7) | Up to 12 | Recruited from hospital testing clinics and referrals | - | 38.11 | - | - | - | - | - | - | 63.89 | - | 6 |
| Rahimi et al., 2024 | Iran | Cross-sectional | 23 | 34.8 | 45.13 (15.32) | 4.2 (mean) | Rehabilitation Research Center | 0 | - | - | - | - | - | - | - | - | - | 6 |
| Holland et al., 2024 | Australia | Cohort | 726 | 35 | 53 (15) | 2 | Tertiary hospital outpatient service | - | - | - | - | - | - | - | - | - | 0.11 | 5 |
| Zalaquett et al., 2024 | US | Cross-sectional | 233 | 16 | 43.2 (-) | - | Community-based healthcare system | - | - | - | - | - | - | - | - | - | 0.152 | 7 |
| Bodey et al., 2024 | UK | Cohort | 112 | 37.5 | 58.5 (-) | 9.8 | Specialist community service | 56.5 | - | - | - | - | - | - | - | - | 0.24 | 4 |
| Kho et al., 2023 | Malaysia | Cohort | 131 | 58.8 | 52 (-) | ~3 | Post-COVID-19 clinic | 100 | 6.1 | 6.1 | 11.5 | 5.3 | 0.8 | - | 27.5 | 0.275 | 0 | 5 |
| Yalcin-Colak et al., 2023 | Turkey | Cross-sectional | 521 | 45.7 | 43.45 (16.91) | 7.43 | University Hospital | 36.9 | - | - | - | - | - | - | 61.2 | 61.2 | 89.8 | 6 |
| Kilincarslan et al. , 2023 | Turkey | Cross-sectional | 266 | 36.7 | 41.2 (11.8) | 3.4 | Tertiary research hospital | 6 | - | - | - | - | - | - | - | - | 89.9 | 8 |

“– “represents the value is not reported by the article.

*represents the age is given in median (IQR)

Table S 3: Sensitivity analysis

| **Sensitivity analysis** | **Pooled health utility value** | **Number of studies** |
| --- | --- | --- |
| Exclude studies with less than 100 samples | 0.77 (0.75, 0.79) | 98 |
| Exclude studies with the category of poor quality (NOS score below 4) | 0.76 [0.74; 0.78] | 125 |
| Exclude studies with the category of poor and moderate quality (NOS score below 6) | 0.77 [0.74; 0.81] | 56 |
| Leave one out meta-analysis | We found that a utility score of 0.77 for 3 studies and 0.76 for the rest with only difference of 0.01 from the mean when omitted one study at a time | |

Table S 4: Predictors of poor health related quality of life in patients with COVID-19

| **author, publication year** | **Predictors of poor health related quality of life** |
| --- | --- |
| Arab-Zozani et al., 2020 | Female, older age, higher education level, being unemployed, ICU admission, having diabetes mellitus, and having heart failure |
| Huang et al, 2021 | Female sex, smoking, and lung diffusion impairment |
| Iqbal et al., 2021 | Disease severity, post-COVID-19 symptoms, comorbidities |
| Kaso et al., 2021 | Elderly, asthma as a comorbidity, general health status on admission (severity), dexamethasone use improve HRQoL |
| Kohlbrenner et al., 2021 | ICU admission |
| Malinowska et al., 2021 | Older age and greater comorbidity |
| Ordinola Navarro et al., 2021 | Altered usual activities and anxiety/depression |
| Shah et al., 2021 | Hospital stay, number of weeks since diagnosis, female gender |
| Todt et al., 2021 | Female sex and intensive care requirement |
| Walle-Hansen et al., 2021 | Severe or critical acute COVID-19, older age, comorbidities |
| Azizi et al., 2022 | Older age, tobacco use, presence of chronic diseases, type 1 diabetes, kidney disease, cardiovascular disease |
| Barani et al., 2022 | Occupation, female, and place of residence |
| Barreto et al., 2022 | Female sex, dyspnea, chest pain, fatigue |
| d’Ettorre et al., 2022 | Female gender, older age, BMI>35, unemployed status, chronic comorbidities |
| Demoule et al., 2022 | Higher body mass index, tracheostomy, male gender, active smoking, dyspnoea on admission to the rehabilitation facility |
| Hegde et al., 2022 | old Age, smoking history |
| Huynh et al, 2022 | Age ≥ 60 years, female, comorbidities, persistent symptoms, living alone, stress |
| Kaso et al., 2022 | Older age, being in worsening condition during admission, having COPD, asthma, and prolonged hospitalization, malignancy |
| Koullias et al., 2022 | Hospitalization, female sex, age 41-60 years, post-COVID-19 Functional Status (PCFS) scale |
| Lim et al., 2022 | Persistent respiratory symptoms |
| Ojeda et al., 2022 | New-onset pain, pain intensity, anxiety, depression |
| Pacho-Hernández et al., 2022 | Sensitization-associated symptoms, depressive levels, anxiety levels, and sleep quality |
| Said et al., 2022 | Seeing a physician for olfactory dysfunction, female gender, chronic pain, depression/anxiety |
| Soh and Cho, 2022 | Female sex, more than three symptoms at baseline, presence of metabolic disease, constitutional symptoms at baseline, neurological symptoms at baseline, neuropsychiatric symptoms at baseline, constitutional symptoms at follow-up, cardiopulmonary symptoms at follow-up, neurological symptoms at follow-up, neuropsychiatric symptoms at follow-up |
| Tak, 2023 | Seeking care in a long COVID clinic, exercising more than five hours per week pre-COVID, experiencing 30 or more symptoms in the previous 30 days |
| Tarazona et al., 2022 | COVID-19 infection, cardiological history, obesity |
| Tsuzuki et al., 2022 | Ongoing prolonged symptoms |
| Umbrello et al., 2022 | Prolonged hospital stay, previous comorbidities |
| Zhang et al., 2022 | Age ≥ 60, female sex, fibrous stripe on chest CT, and having post-COVID-19 symptoms |
| Hoque et al., 2023 | Age (older age >=60), sex (female sex), occupation (housewives and retired individuals), setting( peri-urban, BMI, comorbidities, symptoms (presence of symptom after 1 m) |
| Huarcaya-Victoria et al., 2022 | Age ≥ 41 years, history of psychiatric diagnosis, persistent COVID-19 symptoms, death of a family member from COVID-19 |
| Iribarren-Diarasarri et al., 2022 | Delirium and tracheostomy |
| Román-Montes et al., 2023 | Smoking, severe COVID-19, lower SatO2 on admission, increased lung involvement, and elevated fibrinogen levels |
| Rosa et al., 2023 | severity score |
| Shah et al., 2023 | Age, gender (male), steroid duration, duration of hospital stays, oxygen requirement or ventilation support and its duration, comorbidities, day of illness at admission, severity at admission |
| Wong et al,.2023 | Fatigue, dyspnea, anxiety, and depression (COMBINED) |
| Taboada et al., 2020 | Advanced age, male sex, need for mechanical ventilation, duration of mechanical ventilation, length of ICU stays, and length of hospital stay |
| Ferrarello et al., 2023 | Post-COVID-19 conditions, especially fatigue/exhaustion, headache, memory problems, shortness of breath, and joint or muscle pain |
| Slotegraaf et al, 2023 | Female sex, no hospital admission, lower baseline score |
| Giurgi-Oncu et al., 2021 | Number and intensity of persisting symptoms, cardiovascular and mental health difficulties, pulmonary injury, CRP levels, hospitalization days |
| Sandmann et al., 2021 | Older age, being symptomatic at month 6 |
| Evans et al, 2022 | Female sex, obesity, and invasive mechanical ventilation |
| Brus et al., 2023 | Younger age, female gender, lower level of education, not having paid work before COVID-19, comorbidity, and not being vaccinated. |
| Egger et al., 2024 | Longer duration of mechanical ventilation, Higher preclinical frailty. |
| Neelima et al., 2023 | comorbidities, duration of ICU stay |
| Berentschot J. C, 2024 | Female sex, Unemployment, Pre-existing CVD, Pre-existing pulmonary disease, Longer hospital stay |
| Visser et al., 2024 | Pulmonary embolism during hospitalization |
| Neumann et al., 2025 | At follow-up: Fatigue, number of remaining symptoms, perceived stress, muscle pain, joint pain, and age. |
| Rego de Figueir et al., 2023 | Based on correlation analysis, predictors of QoL index decrease were Age, COPD, asthma, heart failure. |
| Naik et al., 2025 | Lower EQ-5D Utility: Current PCC, severe acute COVID-19, history of depression. |
| Sun X et al., 2024 | Being in a higher symptom burden phenotype was associated with lower HRQoL. |
| Hatakeyama et al., 2025 | For lower EQ-5D-5L score: Age, Delirium, Mechanical ventilation. |
| Soare et al., 2024 | For lower utility score: Acute COVID-19 phase, Long COVID phase, Hospitalisation during acute COVID-19. |
| Tiels eta al., 2025 | lower self-efficacy |
| Mastrorosa I, et al, 2023 | For lower EQ-VAS: Female gender, Presence of comorbidities, Corticosteroid treatment. |
| Mercier et al., 2025 | Post-COVID condition (PCC) |
| van Tol et al., 2024 | Higher frailty at admission |
| Carlile et al., 2024 | Self-reported Long COVID, Disability, Higher number of comorbidities, Lower education, Lower household income |
| Elumalai et al., 2023 | Mobility: Comorbid conditions, Symptomatic COVID. Pain/Discomfort: Comorbid conditions, Symptomatic COVID |
| Di Fusco et al., 2023 | Overall QOL (EQ-5D UI): Being unvaccinated was a predictor of poorer QoL at 6 months compared to being boosted. |
| Kuodi et al., 2023 | Vaccination was associated with a higher QOL score. Hypertension and hospitalization were associated with worse QoL. |
| Walker et al., 2023 | Worse QoL was predicted by higher levels of: Fatigue, Breathlessness, Anxiety, and Depression. |
| Schröder et al., 2024 | Worse QoL was associated with: Long COVID status, female gender, unemployment, heart disease, and psychiatric disorder. |
| Agergaard et al., 2023 | Persistent symptoms after 30 days |
| Caamano E et al., 2024 | Female sex and duration of mechanical ventilation. |
| Bolgeo et al., 2024 | Higher age, greater number of COVID-19 symptoms, and presence of comorbidities at admission. Higher functional impairment at 6 months. |
| Salem et al., 2023 | Older age, Disease severity |
| Macedo Junior c, 2024 | Self-reported dyspnea |
| Álvarez-Hernán, 2023 | Persistent dyspnea. |
| Moisoglou et al., 2024 | Less resilience, less significant other support, longer duration of symptoms. |
| Firouzabadi et a, 2024 | Older age (≥40y), Female gender, ICU admission |
| Sun et al., 2024 | Presence of long-COVID, Low annual household income |
| Galanis et al., 2023 | Longer duration of symptoms; Female gender |
| Dodd et al., 2024 | Long COVID status; Higher CRP; Pre-existing psychological condition |
| Holland et al., 2024 | Long COVID status |
| Kilincarslan et a, 2023 | Female gender, Lower education level, Visual problems, Myalgia |
| Brus et al., 2023 | Younger age, female gender, lower level of education, not having paid work before COVID-19, comorbidity, and not being vaccinated. |
| Egger et al., 2024 | Longer duration of mechanical ventilation, Higher preclinical frailty. |
| Neelima et al., 2023 | comorbidities, duration of ICU stay |
| Berentschot J. C, 2024 | Female sex, Unemployment, Pre-existing CVD, pre-existing pulmonary disease, longer hospital stays |
| Visser et al., 2024 | Pulmonary embolism during hospitalization |
| Neumann et al., 2025 | At follow-up: Fatigue, number of remaining symptoms, perceived stress, muscle pain, joint pain, and age. |
| Rego de Figueir et al., 2023 | Based on correlation analysis, predictors of QoL index decrease were Age, COPD, asthma, heart failure. |
| Naik et al., 2025 | Lower EQ-5D Utility: Current PCC, severe acute COVID-19, history of depression. |
| Sun X et al., 2024 | Being in a higher symptom burden phenotype was associated with lower HRQoL. |
| Hatakeyama et al., 2025 | For lower EQ-5D-5L score: Age, Delirium, Mechanical ventilation. |
| Soare et al., 2024 | For lower utility score: Acute COVID-19 phase, Long COVID phase, Hospitalisation during acute COVID-19. |
| Tiels eta al., 2025 | lower self-efficacy |
| Mastrorosa I, et al, 2023 | For lower EQ-VAS: Female gender, Presence of comorbidities, Corticosteroid treatment. |
| Mercier et al., 2025 | Post-COVID condition (PCC) |
| van Tol et al., 2024 | Higher frailty at admission |
| Carlile et al., 2024 | Self-reported Long COVID, Disability, Higher number of comorbidities, Lower education, Lower household income |
| Elumalai et al., 2023 | Mobility: Comorbid conditions, Symptomatic COVID. Pain/Discomfort: Comorbid conditions, Symptomatic COVID |
| Di Fusco et al., 2023 | Overall QOL (EQ-5D UI): Being unvaccinated was a predictor of poorer QoL at 6 months compared to being boosted. |
| Kuodi et al., 2023 | Vaccination was associated with a higher QOL score. Hypertension and hospitalization were associated with worse QoL. |
| Walker et al., 2023 | Worse QoL was predicted by higher levels of: Fatigue, Breathlessness, Anxiety, and Depression. |
| Schröder et al., 2024 | Worse QoL was associated with: Long COVID status, female gender, unemployment, heart disease, and psychiatric disorder. |
| Agergaard et al., 2023 | Persistent symptoms after 30 days |
| Caamano E et al., 2024 | Female sex and duration of mechanical ventilation. |
| Bolgeo et al., 2024 | Higher age, greater number of COVID-19 symptoms, and presence of comorbidities at admission. Higher functional impairment at 6 months. |
| Salem et al., 2023 | Older age, Disease severity |
| Macedo Junior c, 2024 | Self-reported dyspnea |
| Álvarez-Hernán, 2023 | Persistent dyspnea. |
| Moisoglou et al., 2024 | Less resilience, less significant other support, longer duration of symptoms. |
| Firouzabadi et a, 2024 | Older age (≥40y), Female gender, ICU admission |
| Sun et al., 2024 | Presence of long-COVID, Low annual household income |
| Galanis et al., 2023 | Longer duration of symptoms; Female gender |
| Dodd et al., 2024 | Long COVID status; Higher CRP; Pre-existing psychological condition |
| Holland et al., 2024 | Long COVID status |
| Kilincarslan et a, 2023 | Female gender, Lower education level, Visual problems, Myalgia |


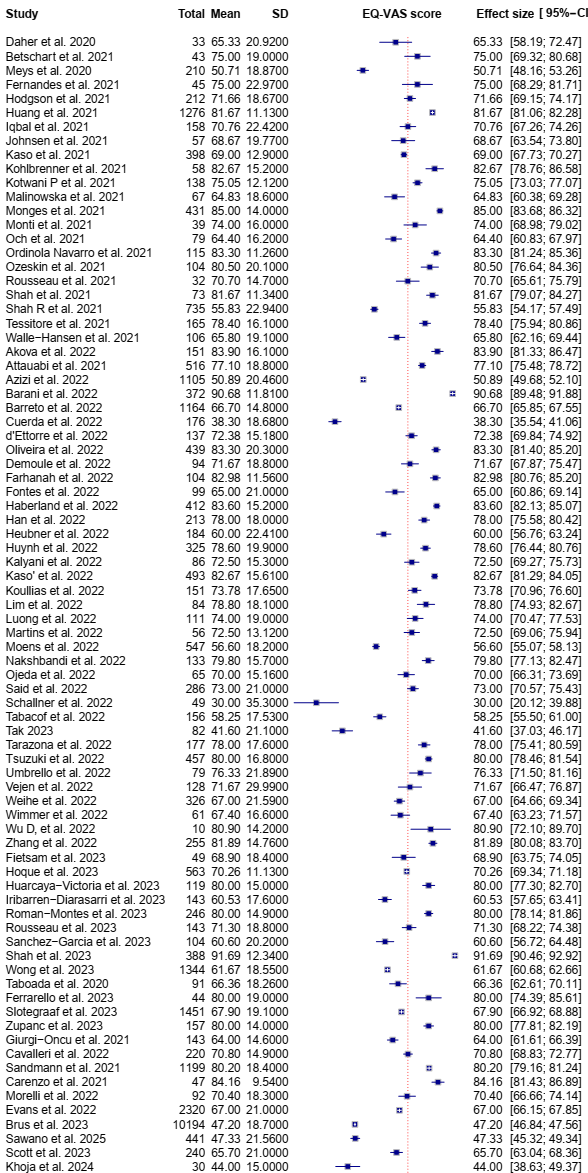


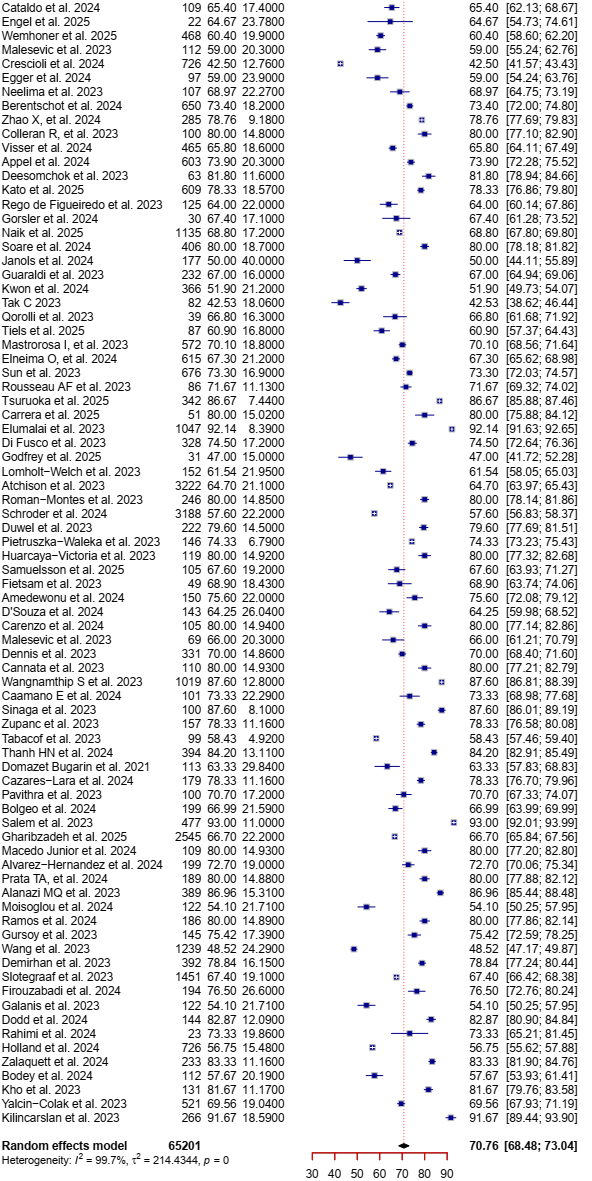


Figure S 1: Random effect meta-analysis of EQ-VAS score in patients with COVID-19


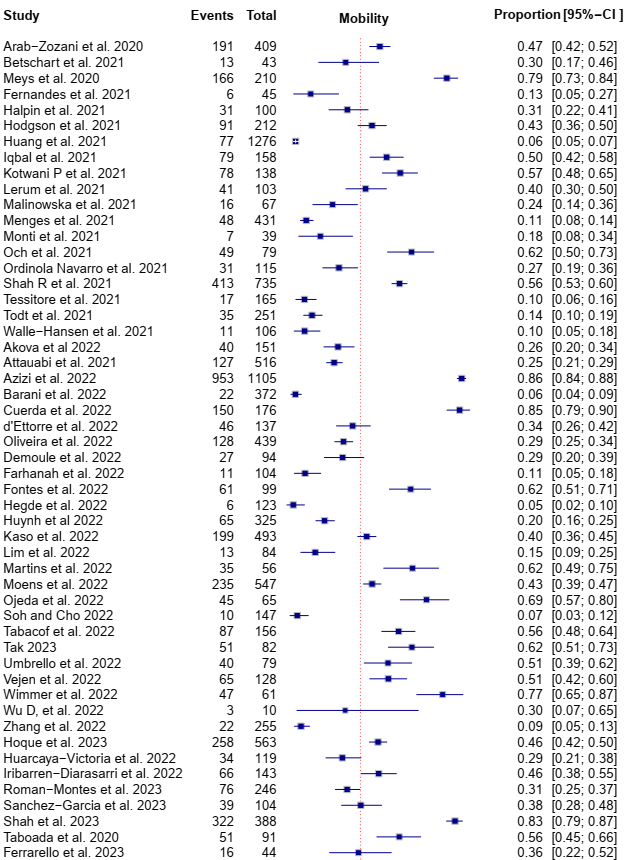


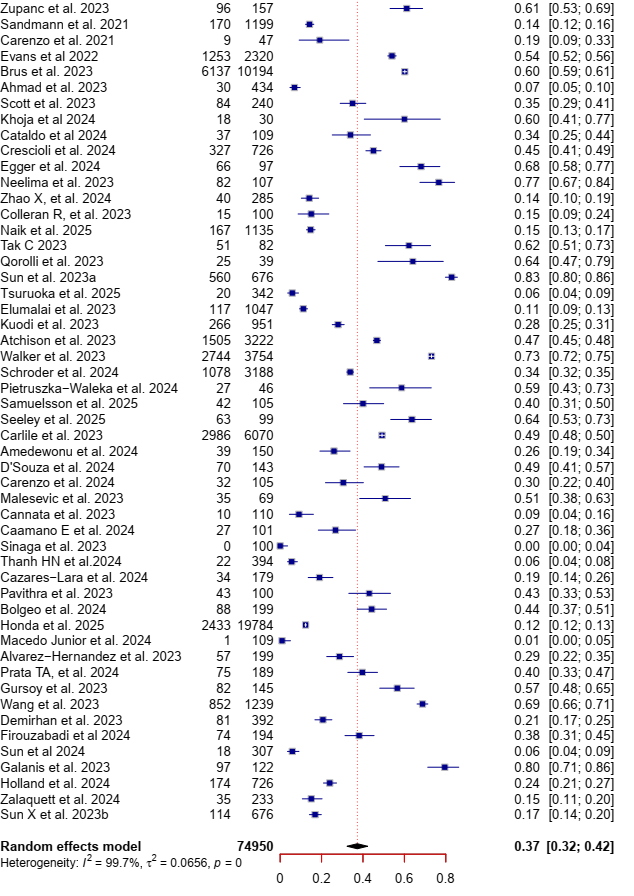
 Figure S 2: Forest plot of pooled proportion of mobility using EQ‐5D in COVID‐19 patients.


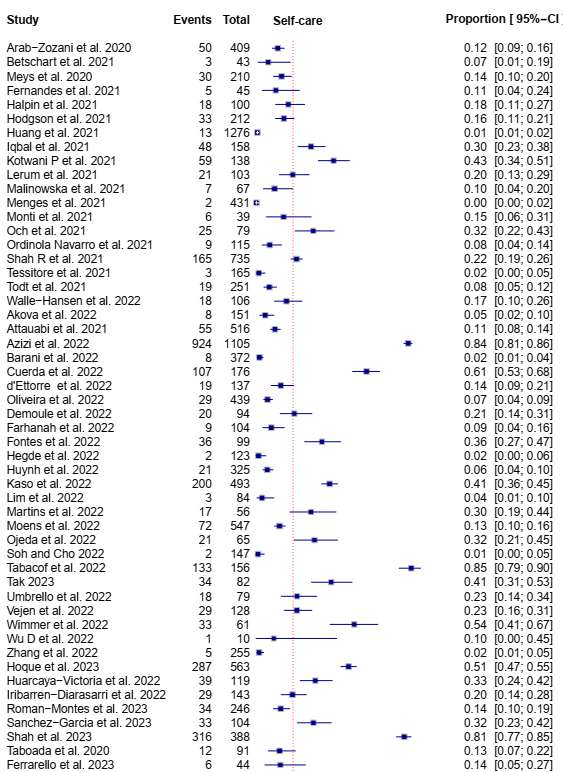


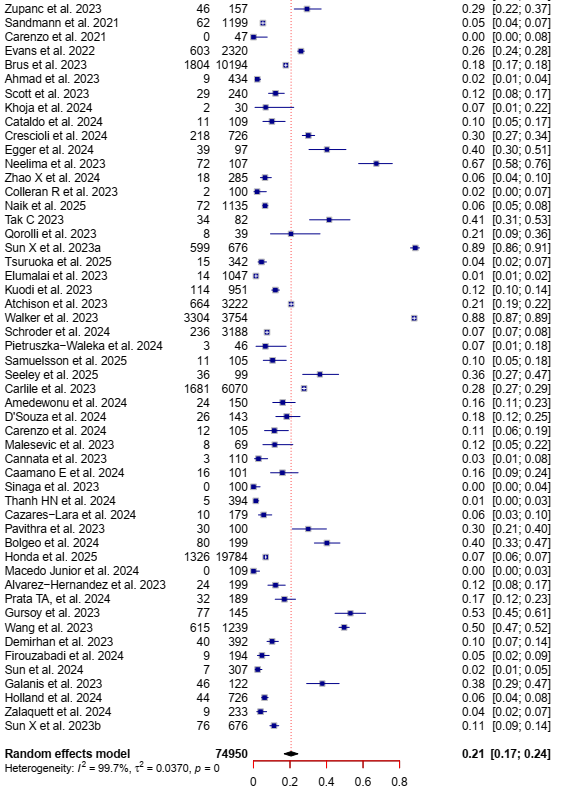


Figure S 3: Forest plot of pooled proportion of self-care using EQ‐5D in COVID‐19 patients.


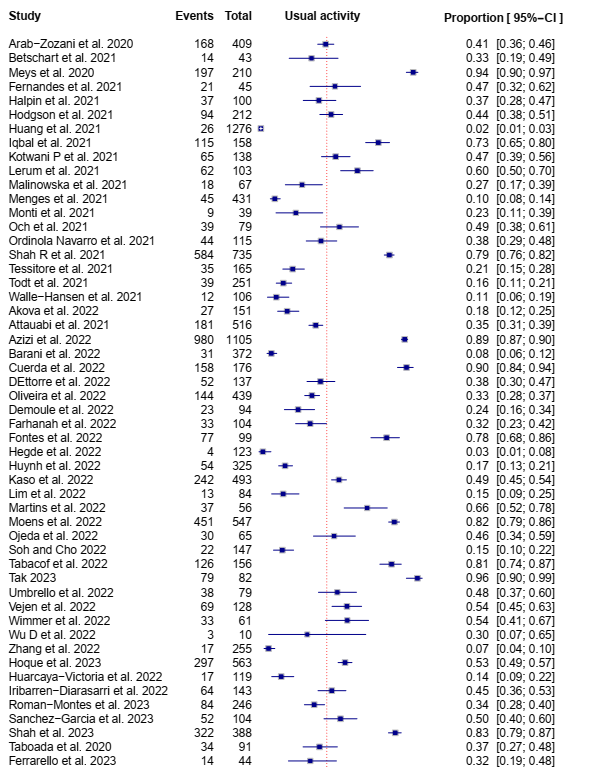


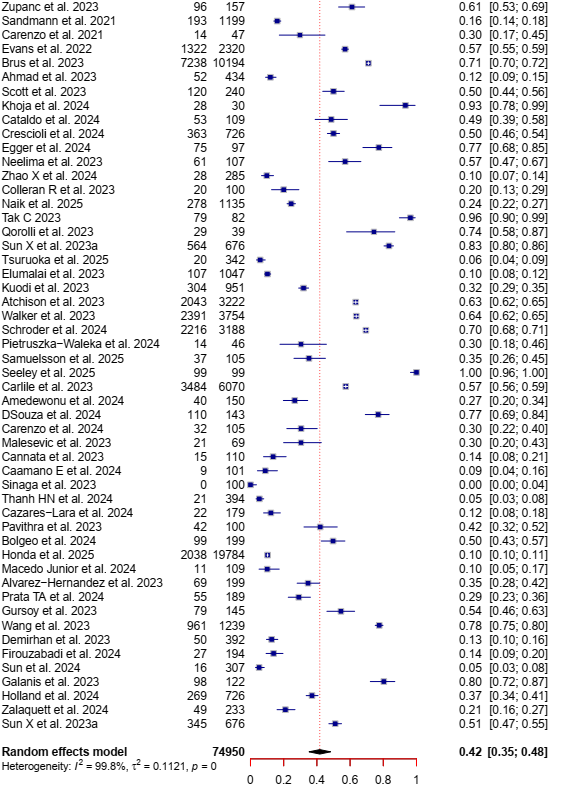

Figure S 4: Forest plot of pooled proportion of usual activity using EQ‐5D in COVID‐19 patients.


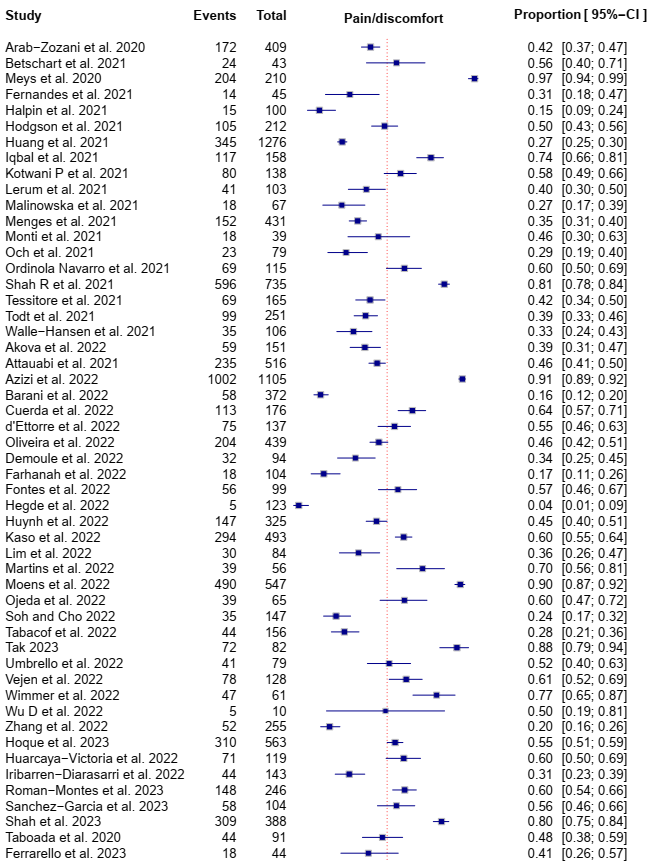


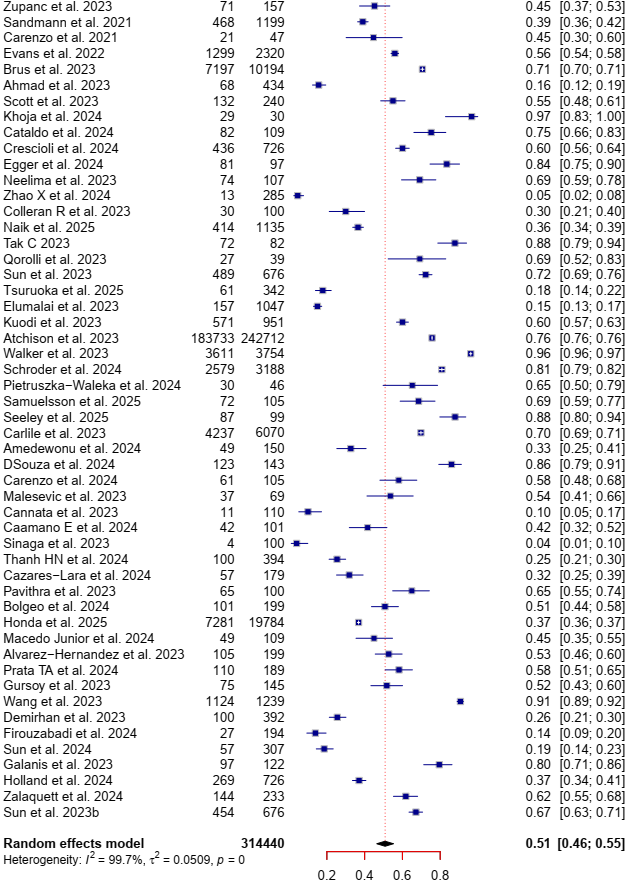


Figure S 5: Forest plot of pooled proportion of pain/discomfort using EQ‐5D in COVID‐19 patients.


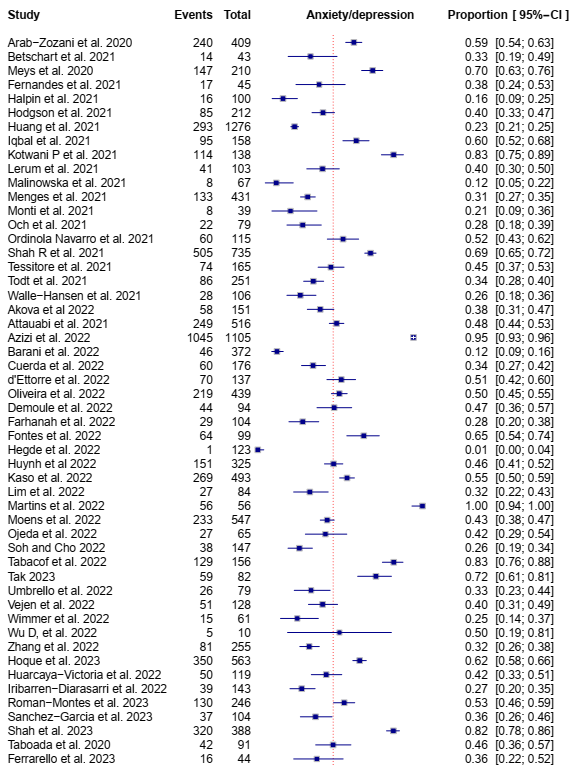


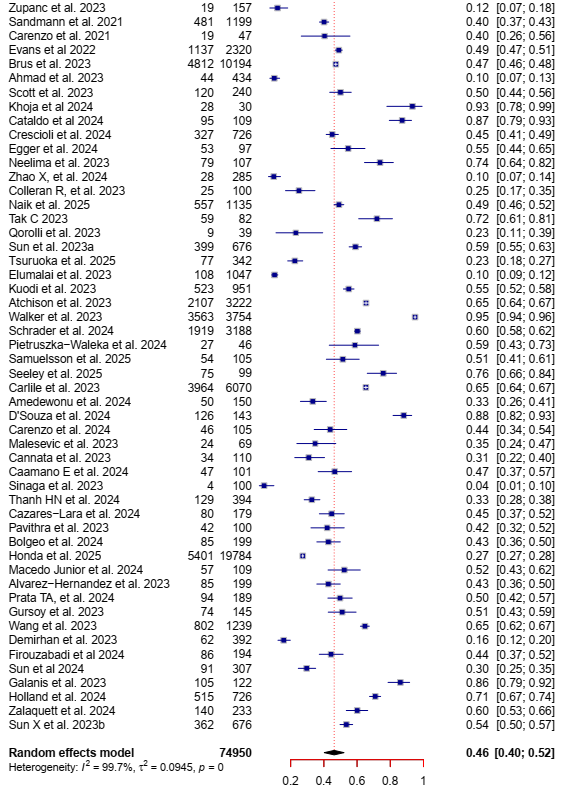


Figure S 6: Forest plot of pooled proportion of anxiety/depression using EQ‐5D in COVID‐19 patients.


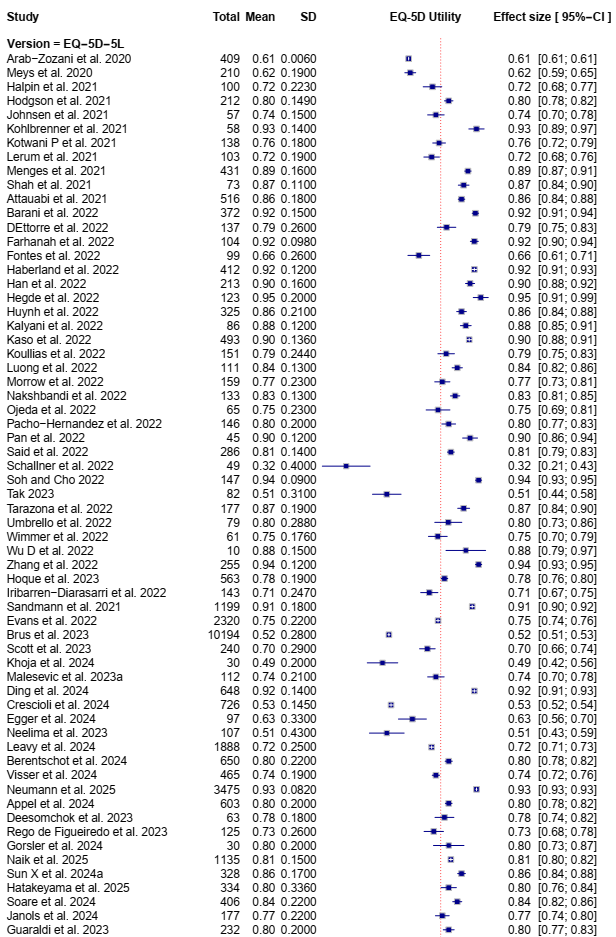


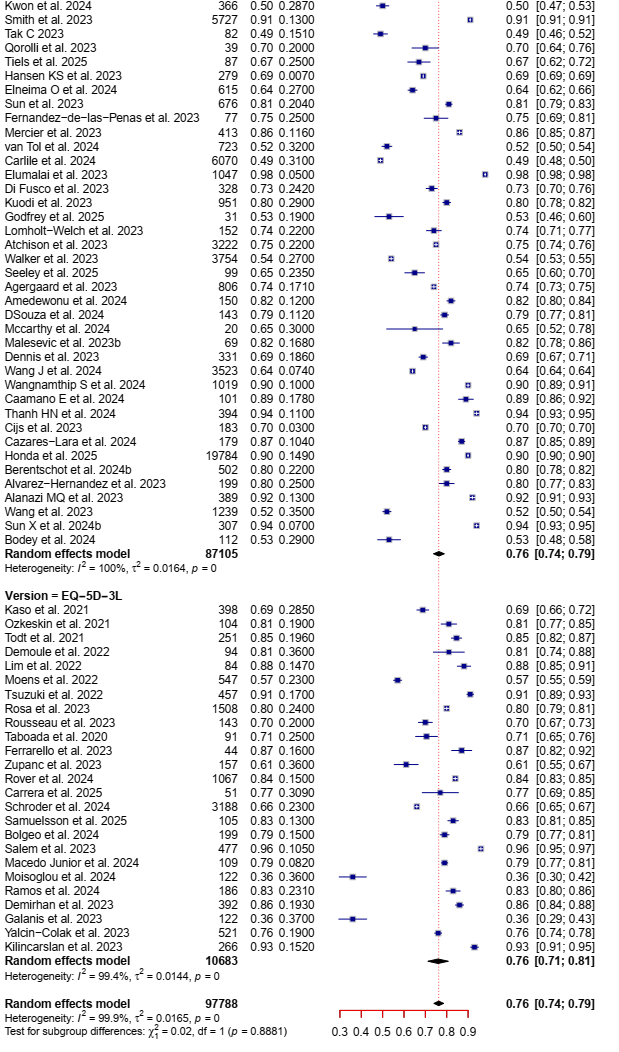


Figure S 7: EQ-5D instrument versions stratified random effect meta-analysis


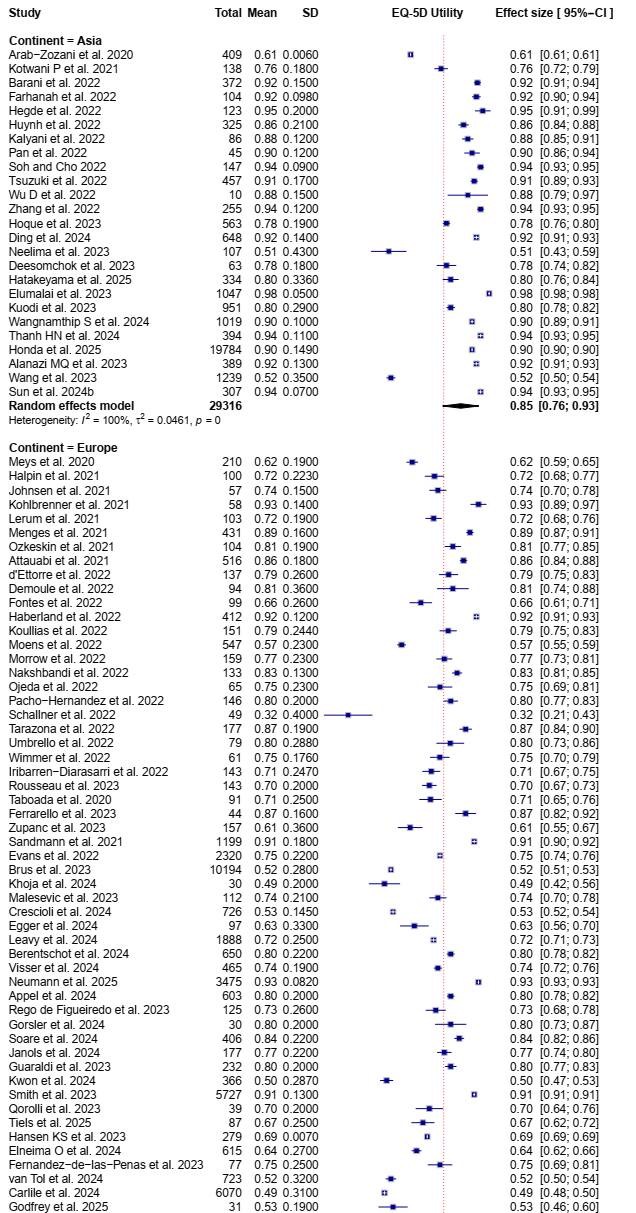


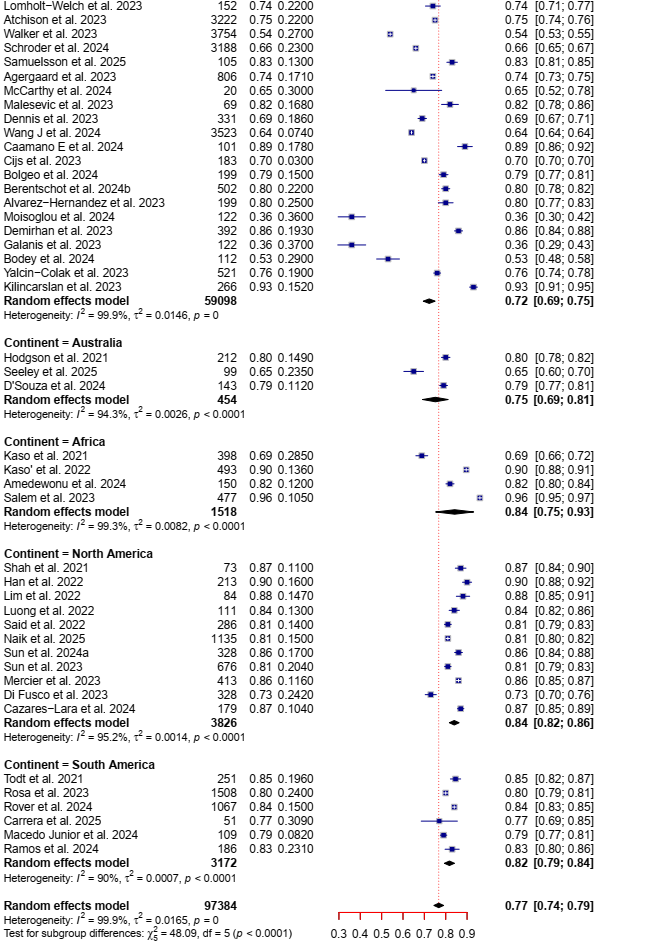


Figure S 8: Geographic locations stratified random effect meta-analysis


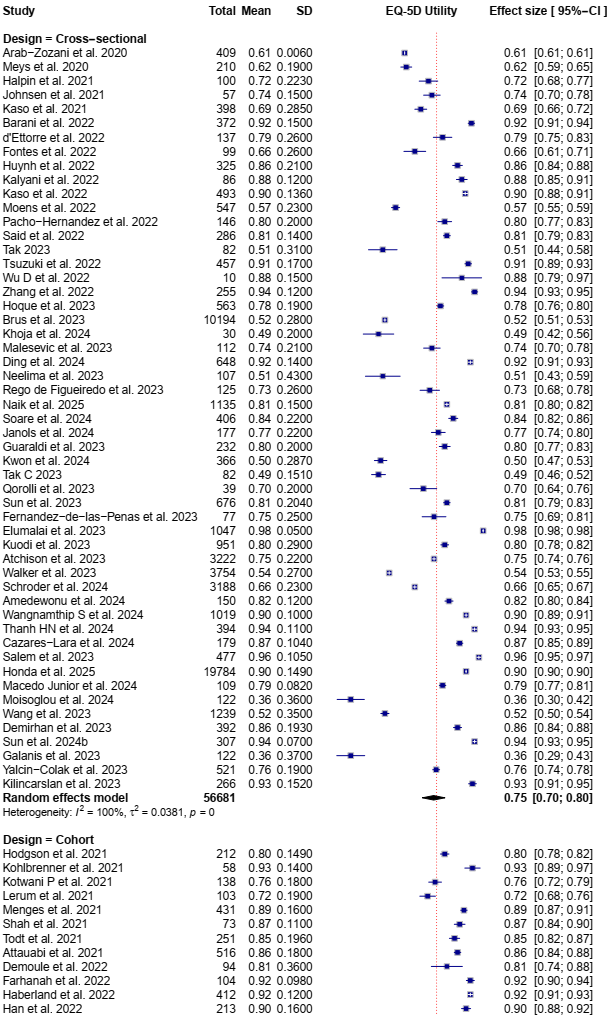


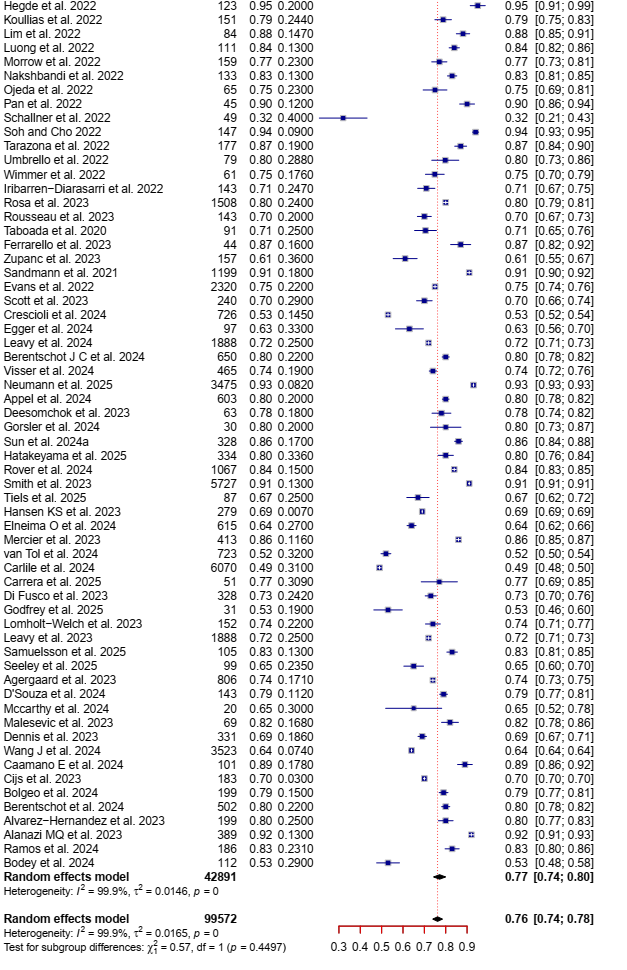


Figure S 9: Study design stratified random effect meta-analysis


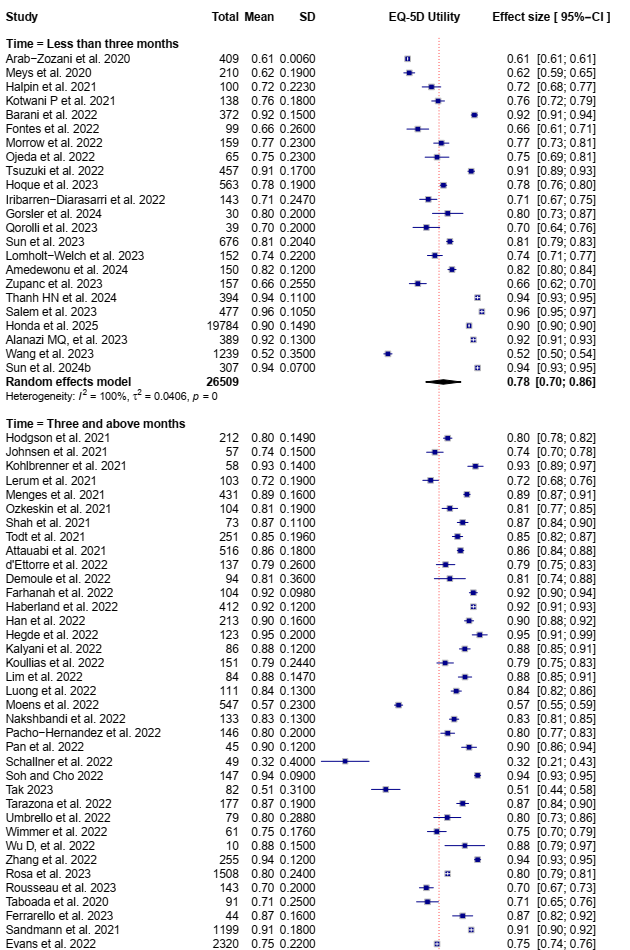

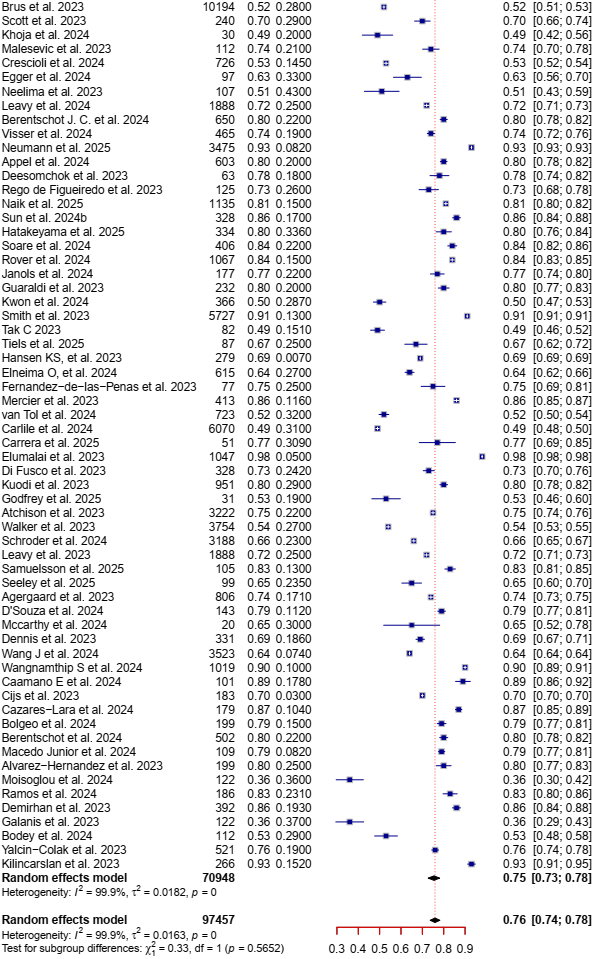


Figure S 10: Time of HRQoL measurements after the COVID-19 diagnosis stratified random effect meta-analysis


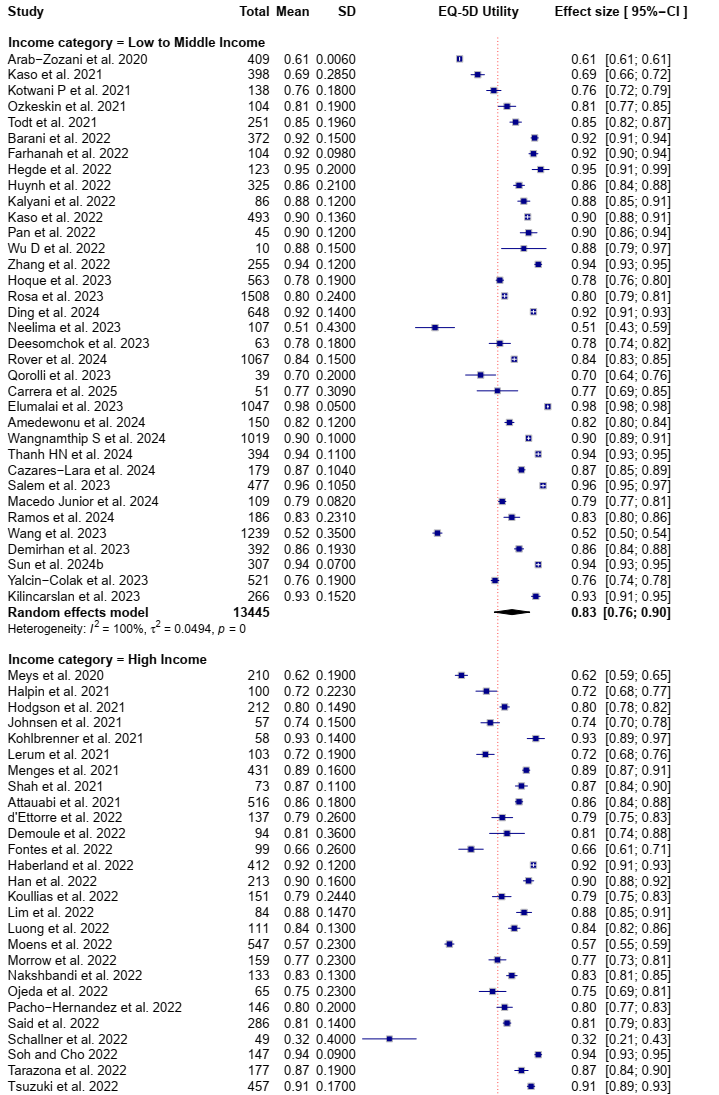


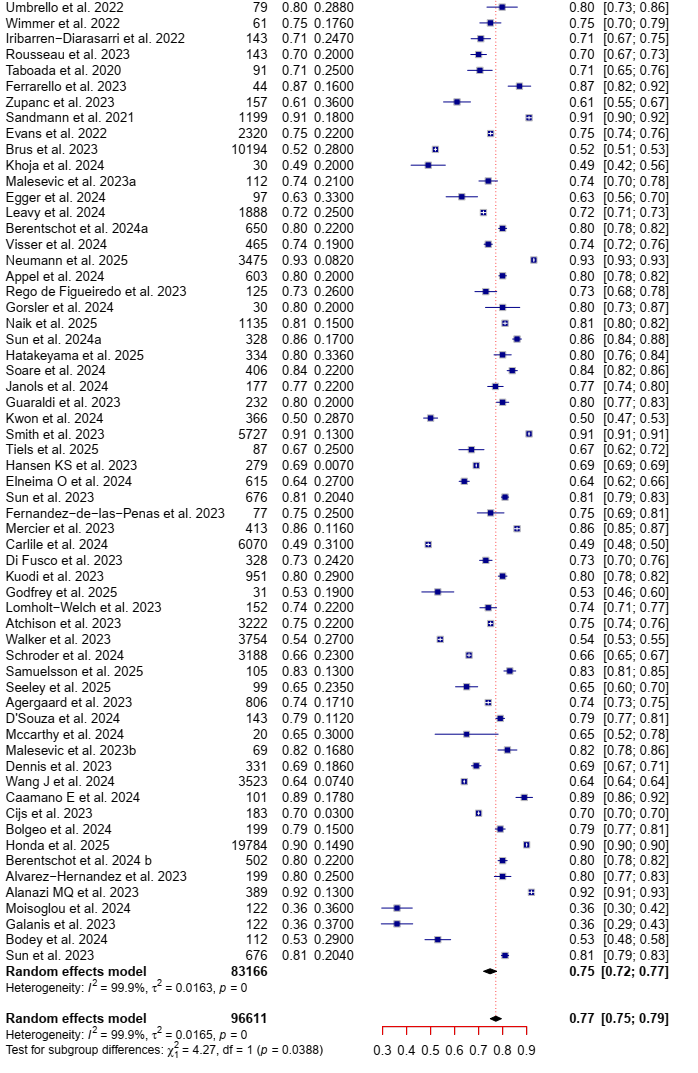


Figure S 11: Income category stratified random effect meta-analysis


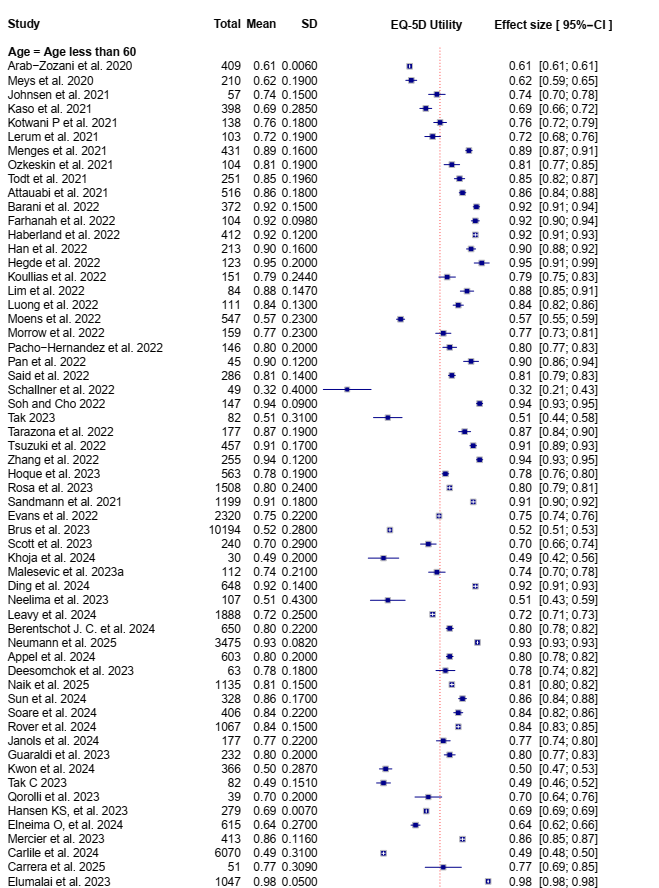


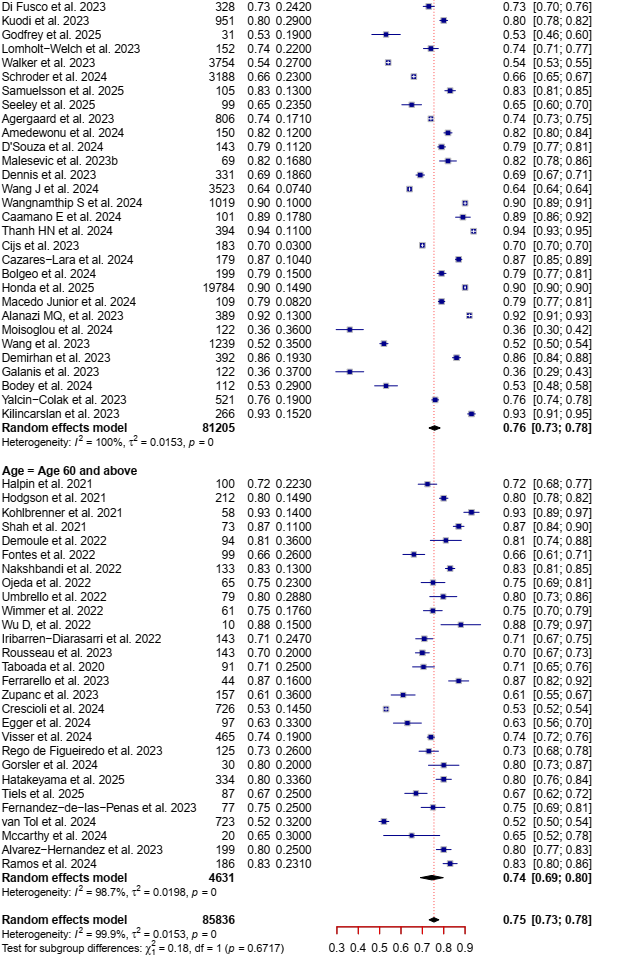


Figure S 12: Age group stratified random effect meta-analysis


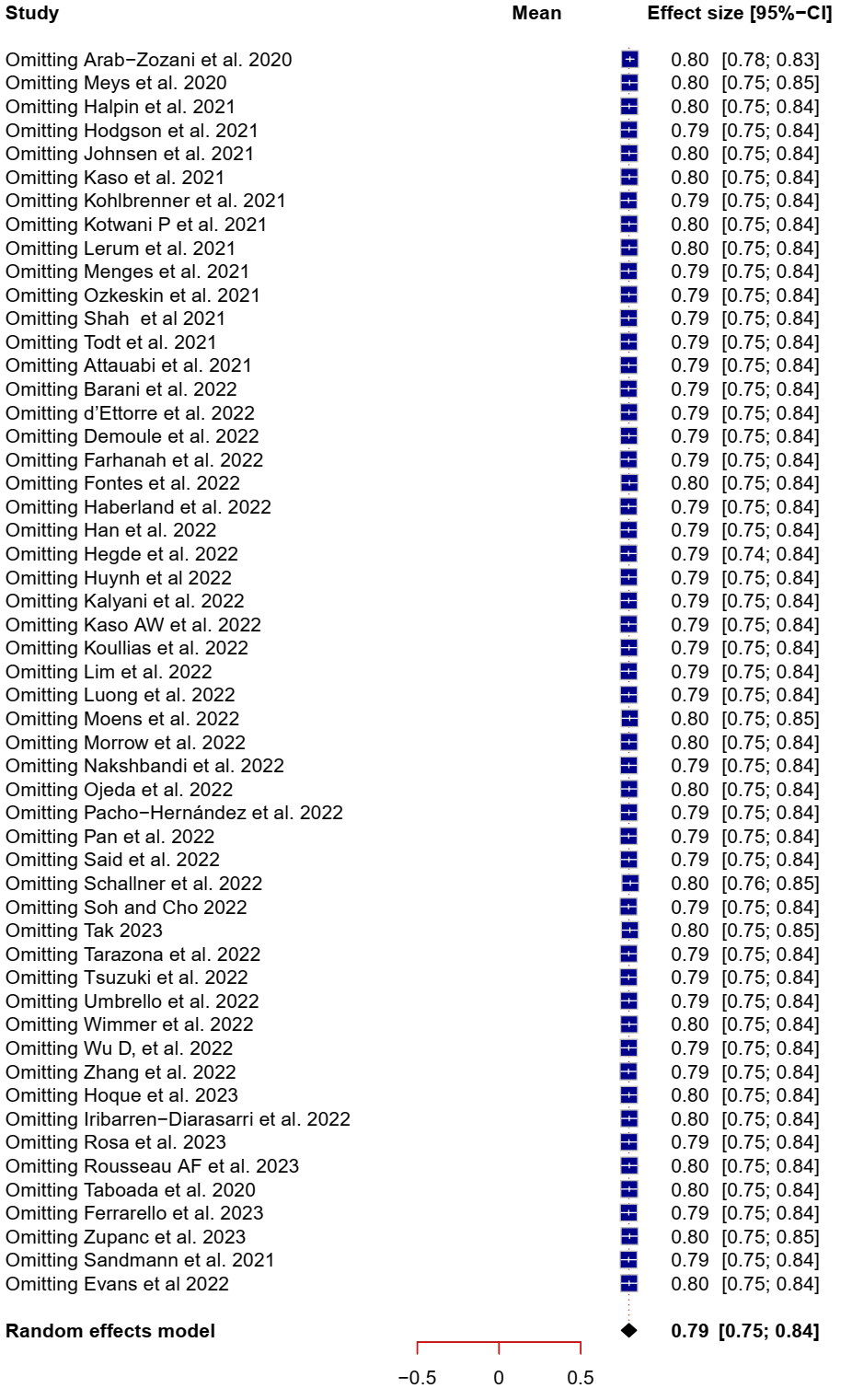
\

Figure S 13: Sensitivity analysis using the leave-one-out meta-analysis method

Appendix 1: PRISMA checklist

| **Section and Topic** | **Item #** | **Checklist item** | **Location where item is reported** |
| --- | --- | --- | --- |
| **TITLE** | | |  |
| Title | 1 | Identify the report as a systematic review. | Page 1 |
| **ABSTRACT** | | |  |
| Abstract | 2 | See the PRISMA 2020 for Abstracts checklist. | Reviewed (compliant) |
| **INTRODUCTION** | | |  |
| Rationale | 3 | Describe the rationale for the review in the context of existing knowledge. | Page 3-4 |
| Objectives | 4 | Provide an explicit statement of the objective(s) or question(s) the review addresses. | Page 5 |
| **METHODS** | | |  |
| Eligibility criteria | 5 | Specify the inclusion and exclusion criteria for the review and how studies were grouped for the syntheses. | Page 5-6 |
| Information sources | 6 | Specify all databases, registers, websites, organisations, reference lists and other sources searched or consulted to identify studies. Specify the date when each source was last searched or consulted. | Page 5 |
| Search strategy | 7 | Present the full search strategies for all databases, registers and websites, including any filters and limits used. | Table S1 |
| Selection process | 8 | Specify the methods used to decide whether a study met the inclusion criteria of the review, including how many reviewers screened each record and each report retrieved, whether they worked independently, and if applicable, details of automation tools used in the process. | Page 6-7 |
| Data collection process | 9 | Specify the methods used to collect data from reports, including how many reviewers collected data from each report, whether they worked independently, any processes for obtaining or confirming data from study investigators, and if applicable, details of automation tools used in the process. | Page 7 |
| Data items | 10a | List and define all outcomes for which data were sought. Specify whether all results that were compatible with each outcome domain in each study were sought (e.g. for all measures, time points, analyses), and if not, the methods used to decide which results to collect. | Page 7 |
|  | 10b | List and define all other variables for which data were sought (e.g. participant and intervention characteristics, funding sources). Describe any assumptions made about any missing or unclear information. | Page 7 |
| Study risk of bias assessment | 11 | Specify the methods used to assess risk of bias in the included studies, including details of the tool(s) used, how many reviewers assessed each study and whether they worked independently, and if applicable, details of automation tools used in the process. | Page 6 |
| Effect measures | 12 | Specify for each outcome the effect measure(s) (e.g. risk ratio, mean difference) used in the synthesis or presentation of results. | Page 8 |
| Synthesis methods | 13a | Describe the processes used to decide which studies were eligible for each synthesis (e.g. tabulating the study intervention characteristics and comparing against the planned groups for each synthesis (item #5)). | Page 8 |
|  | 13b | Describe any methods required to prepare the data for presentation or synthesis, such as handling of missing summary statistics, or data conversions. | Page 8 |
|  | 13c | Describe any methods used to tabulate or visually display results of individual studies and syntheses. | Page 8 |
|  | 13d | Describe any methods used to synthesize results and provide a rationale for the choice(s). If meta-analysis was performed, describe the model(s), method(s) to identify the presence and extent of statistical heterogeneity, and software package(s) used. | Page 8 |
|  | 13e | Describe any methods used to explore possible causes of heterogeneity among study results (e.g. subgroup analysis, meta-regression). | Page 8 |
|  | 13f | Describe any sensitivity analyses conducted to assess robustness of the synthesized results. | Page 8 |
| Reporting bias assessment | 14 | Describe any methods used to assess risk of bias due to missing results in a synthesis (arising from reporting biases). | Page 8 |
| Certainty assess | 15 | Describe any methods used to assess certainty (or confidence) in the body of evidence for an outcome. | Page 8 |
| **RESULTS** | | |  |
| Study selection | 16a | Describe the results of the search and selection process, from the number of records identified in the search to the number of studies included in the review, ideally using a flow diagram. | Page 9 |
|  | 16b | Cite studies that might appear to meet the inclusion criteria, but which were excluded, and explain why they were excluded. | Page 9 |
| Study characteristics | 17 | Cite each included study and present its characteristics. | Table S1 |
| Risk of bias in studies | 18 | Present assessments of risk of bias for each included study. | Table s1 |
| Results of individual studies | 19 | For all outcomes, present, for each study: (a) summary statistics for each group (where appropriate) and (b) an effect estimate and its precision (e.g. confidence/credible interval), ideally using structured tables or plots. | Table 2 |
| Results of syntheses | 20a | For each synthesis, briefly summarise the characteristics and risk of bias among contributing studies. | Table S1 |
|  | 20b | Present results of all statistical syntheses conducted. If meta-analysis was done, present for each the summary estimate and its precision (e.g. confidence/credible interval) and measures of statistical heterogeneity. If comparing groups, describe the direction of the effect. | Page 9-12 |
|  | 20c | Present results of all investigations of possible causes of heterogeneity among study results. | Page 11-14 |
|  | 20d | Present results of all sensitivity analyses conducted to assess the robustness of the synthesized results. | Page 15 |
| Reporting biases | 21 | Present assessments of risk of bias due to missing results (arising from reporting biases) for each synthesis assessed. | page 17 |
| Certainty of evidence | 22 | Present assessments of certainty (or confidence) in the body of evidence for each outcome assessed. | Page 15 |
| **DISCUSSION** | | |  |
| Discussion | 23a | Provide a general interpretation of the results in the context of other evidence. | Page 18-23 |
|  | 23b | Discuss any limitations of the evidence included in the review. | Page 22 |
|  | 23c | Discuss any limitations of the review processes used. | Page 22 |
|  | 23d | Discuss implications of the results for practice, policy, and future research. | Page 18-22 |
| **OTHER INFORMATION** | | |  |
| Registration and protocol | 24a | Provide registration information for the review, including register name and registration number, or state that the review was not registered. | Page 5 |
|  | 24b | Indicate where the review protocol can be accessed, or state that a protocol was not prepared. | - |
|  | 24c | Describe and explain any amendments to information provided at registration or in the protocol. | - |
| Support | 25 | Describe sources of financial or non-financial support for the review, and the role of the funders or sponsors in the review. | Page 24 |
| Competing interests | 26 | Declare any competing interests of review authors. | Page 24 |
| Availability of data, code and other materials | 27 | Report which of the following are publicly available and where they can be found: template data collection forms; data extracted from included studies; data used for all analyses; analytic code; any other materials used in the review. | Page 24 |

Appendix 2: Format for the quality assessment of the articles

**Cohort studies**

1. Selection (Maximum 4 scores)
   1. Representativeness of the exposed cohort, if yes 1 score if no 0 score
   2. Selection of the non-exposed cohort [Evaluate if the comparison group (non-COVID-19 patients or general population) is appropriate and if it is representative of the target population] , if yes 1 score if no 0 score
   3. Ascertainment of exposure [The exposure of interest, which is COVID-19, was confirmed by laboratory tests and clinical criteria] , if yes 1 score if no 0 score
   4. Demonstration that outcome of interest was not present at the start of the study (Determine if the study participants did not have the outcome of interest (e.g., poor health-related quality of life) at the beginning of the study), if yes 1 score if no 0 score
2. Comparability (Maximum 2 scores)
   1. Study controls for most important factor [age and sex] , if yes 1 score if no 0 score
   2. Study controls for an additional factor [other factors] , if yes 1 score if no 0 score
3. Outcome (Maximum 3 scores)
   1. Assessment of outcome [used eq 5d instrument] , if yes 1 score if no 0 score
   2. Was follow-up long enough for outcomes to occur, if yes 1 score if no 0 score
   3. Adequacy of follow up of cohorts [Adequate follow-up was defined as ≥80% attendance at all follow-up intervals] , if yes 1 score if no 0 score

**Case control studies**

1. Selection (Maximum 4 scores)
   1. Adequate case definition, if yes 1 score if no 0 score
   2. Representativeness of the cases , if yes 1 score if no 0 score
   3. Selection of Controls (community control) , if yes 1 score if no 0 score
   4. Definition of Controls (no history of disease) , if yes 1 score if no 0 score
2. Comparability (Maximum 2 scores)
   1. Study controls for most important factor [age and sex] , if yes 1 score if no 0 score
   2. Study controls for an additional factor [other factors] , if yes 1 score if no 0 score
3. Outcome (Maximum 3 scores)
   1. Ascertainment of exposure [The exposure of interest, which is COVID-19, was confirmed by laboratory tests and clinical criteria] , if yes 1 score if no 0 score
   2. The same method of ascertainment for cases and controls, if yes 1 score if no 0 score
   3. the same non-response rate for both groups, if yes 1 score if no 0 score

**Cross sectional studies**

1. selection (Maximum 4 scores)
   1. Representativeness of the sample, if yes 1 score if no 0 score
   2. Sample size [sample size is justified and > 100] , if yes 1 score if no 0 score
   3. The response rate [ response rate > 80] , if yes 1 score if no 0 score
   4. Ascertainment of the exposure [The exposure of interest, which is COVID-19, was confirmed by laboratory tests and clinical criteria] , if yes 1 score if no 0 score
2. Comparability (Maximum 2 scores)
   1. Study controls for most important factor [age and sex] , if yes 1 score if no 0 score
   2. Study controls for an additional factor [other factors] , if yes 1 score if no 0 score
3. Outcome (Maximum 3 scores)
   1. Assessment of the outcome [Evaluate if the measurement tool used for assessing HRQoL is valid and reliable] , if yes 2 score if no 0 score
   2. The statistical test used to analyze the data is clearly described and appropriate, and the measurement of the association is presented, including confidence intervals and the probability level (p value). , if yes 1 score if no 0 score
